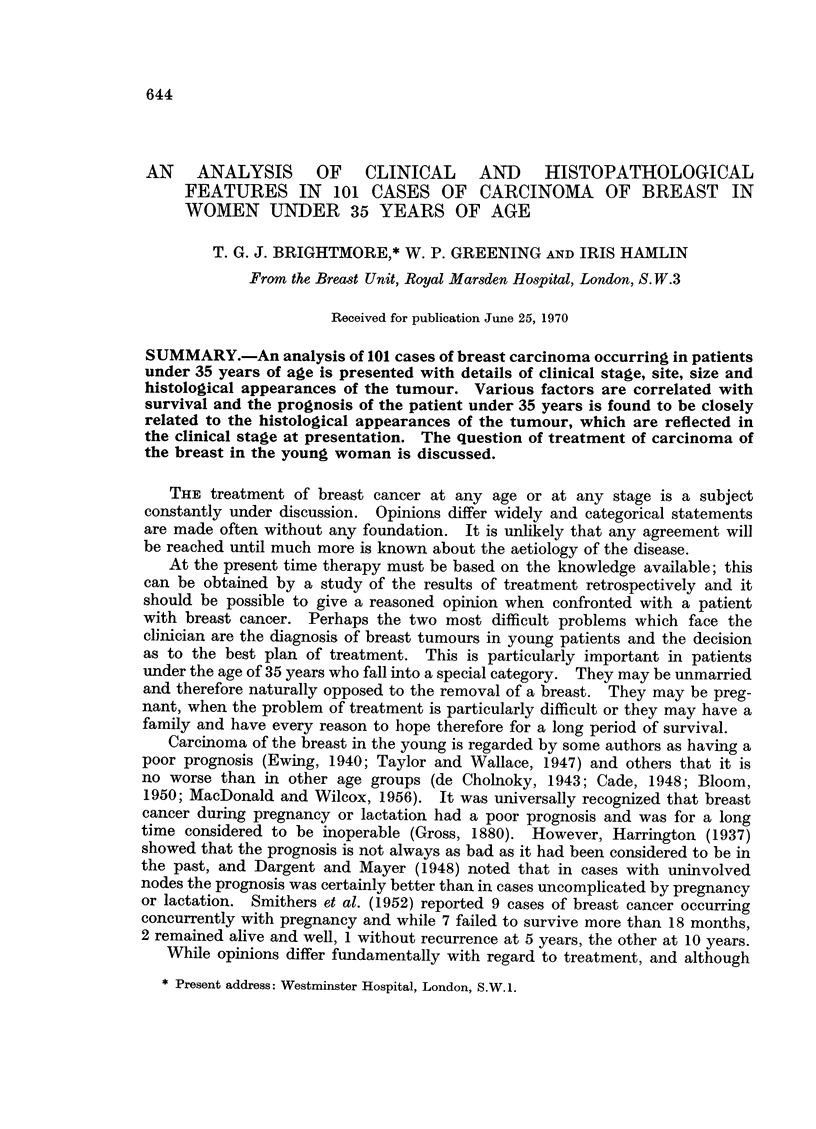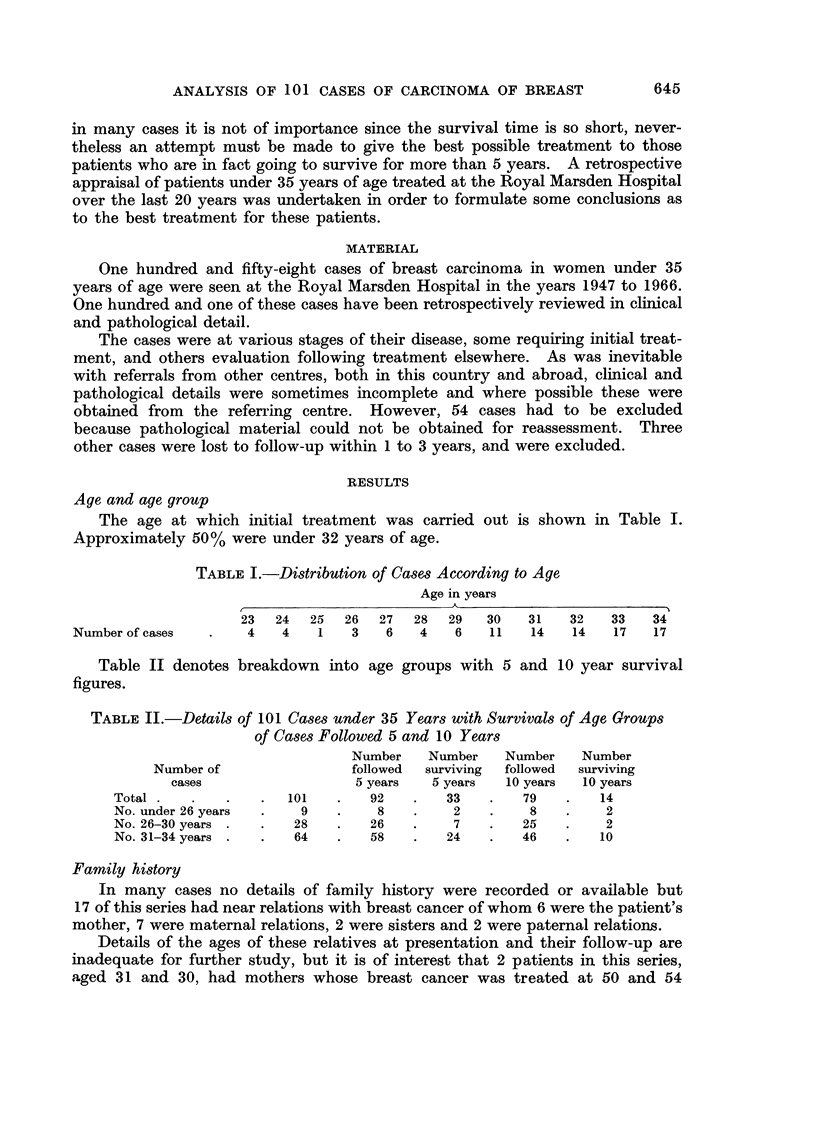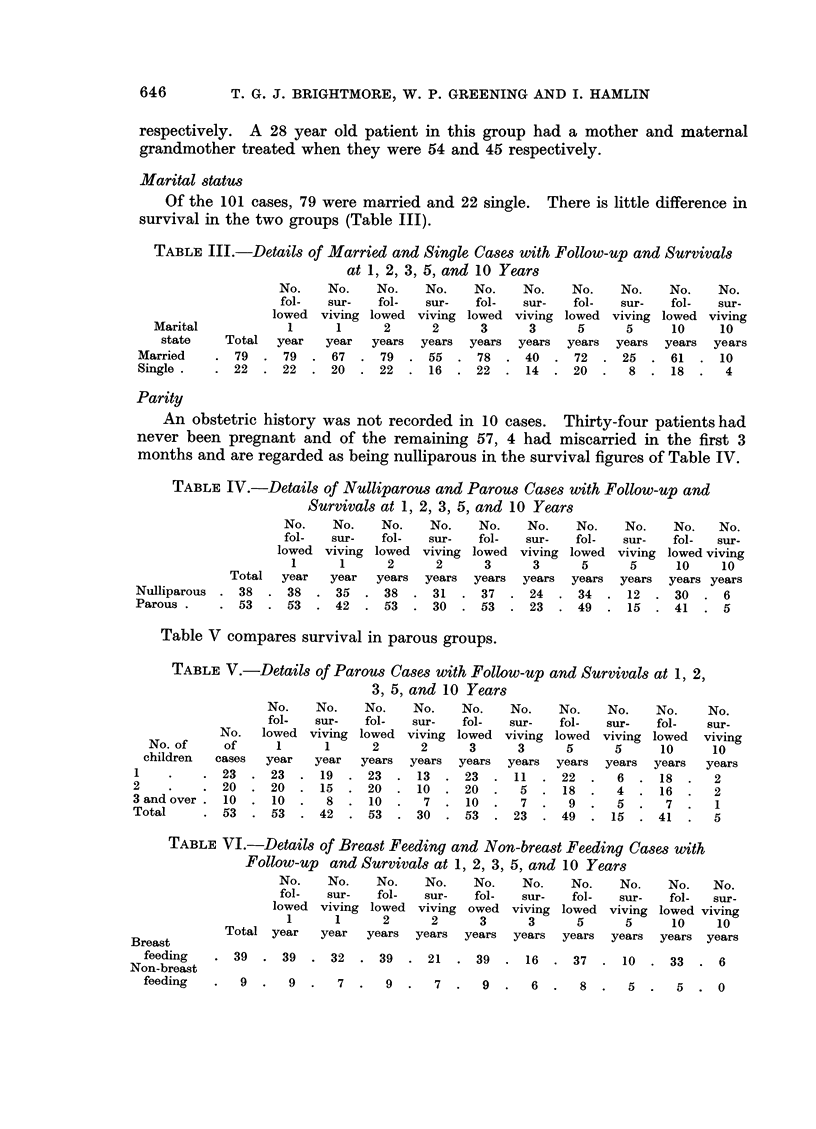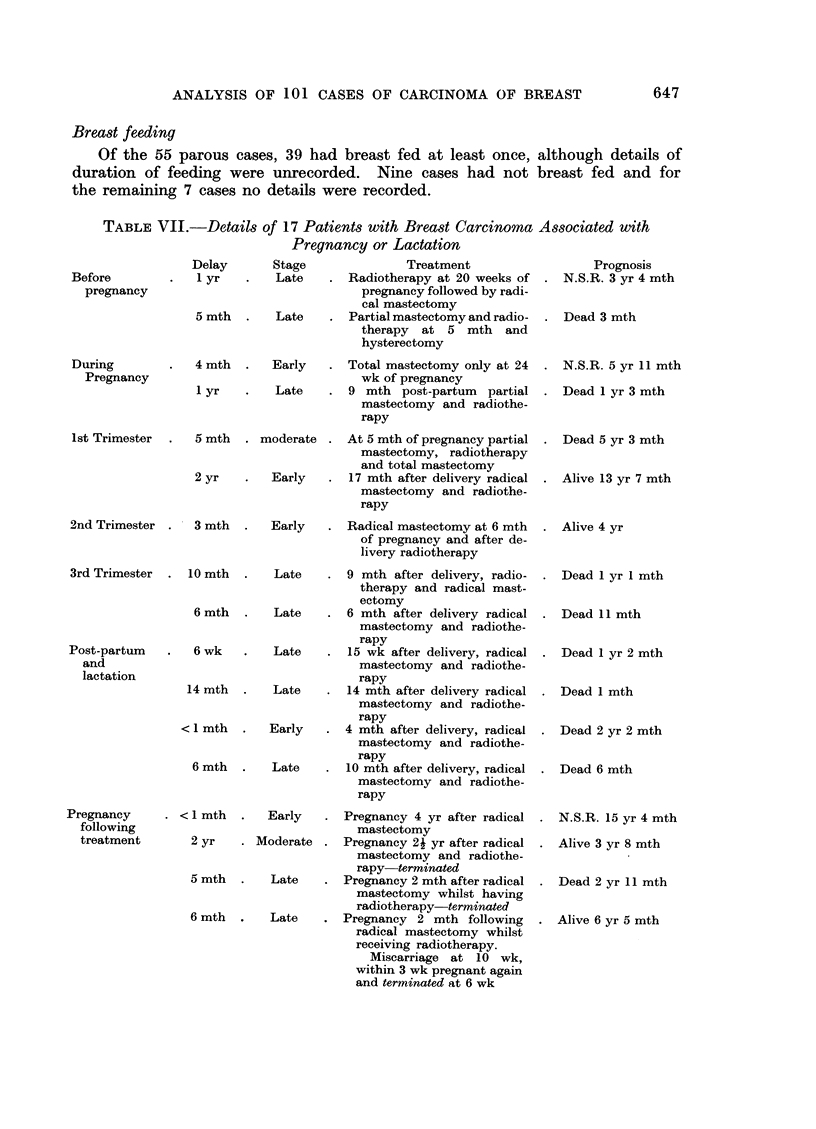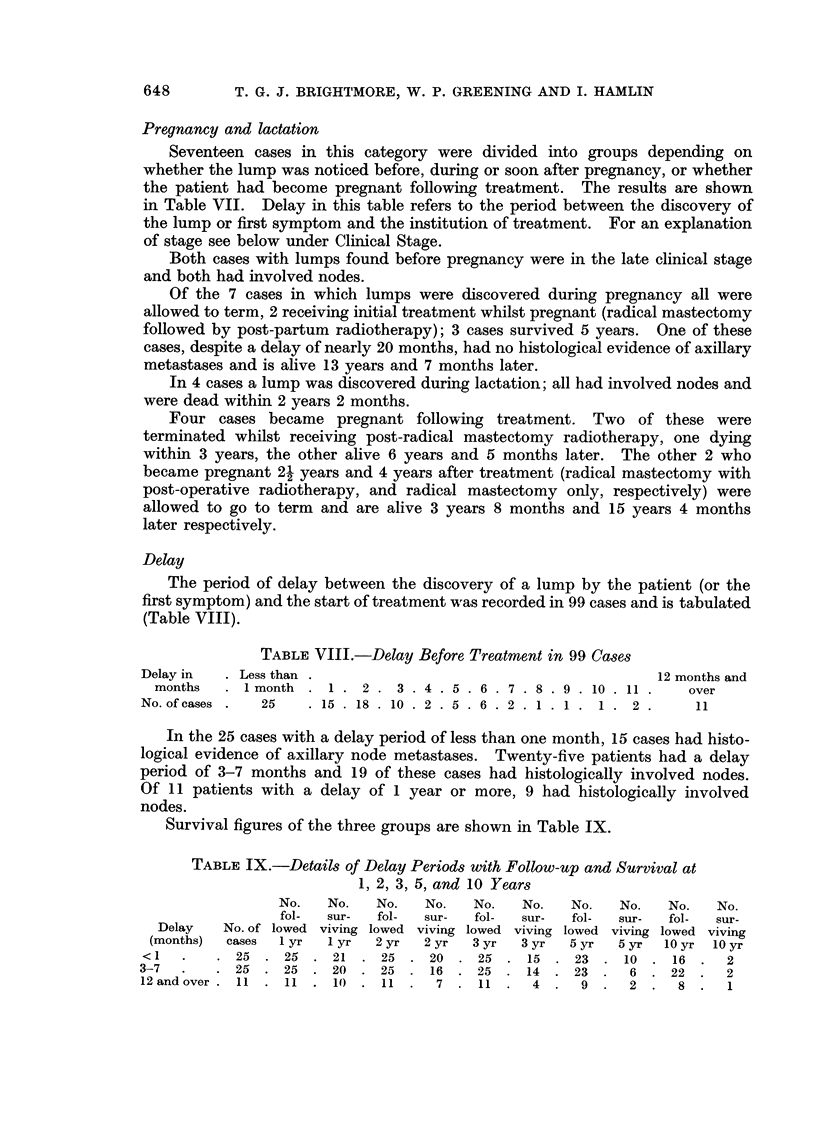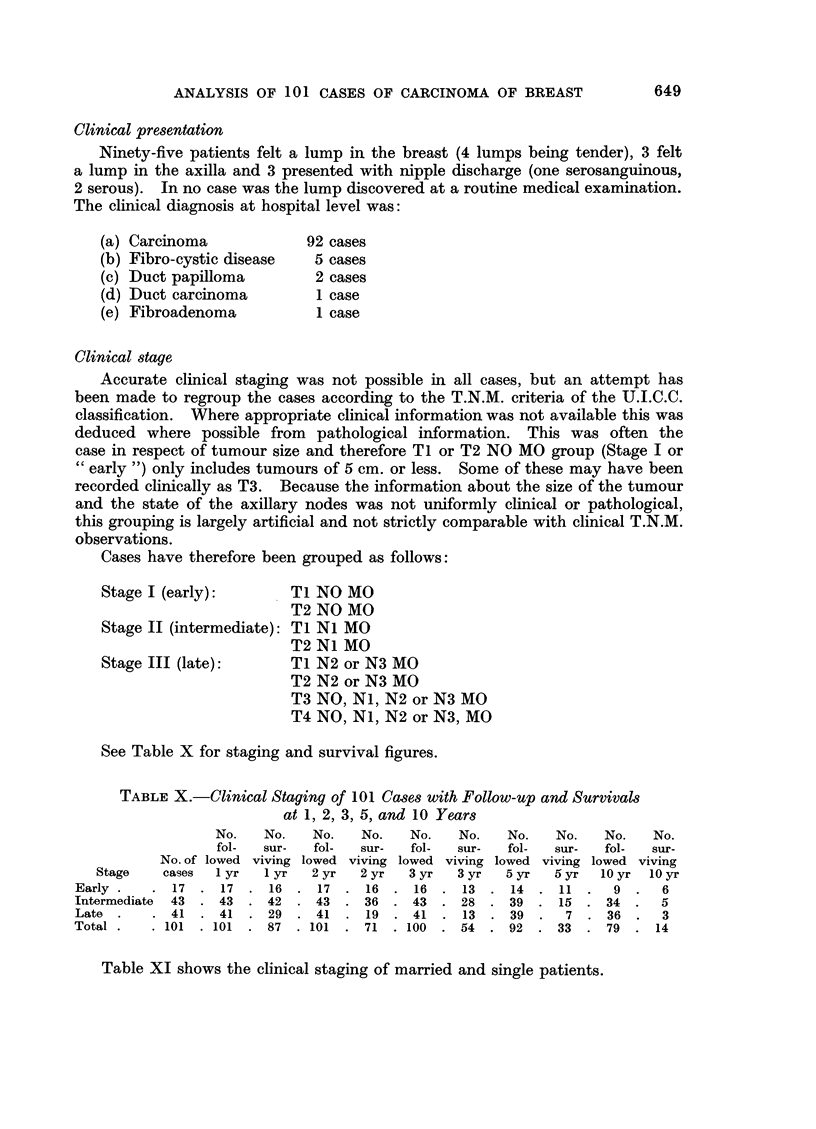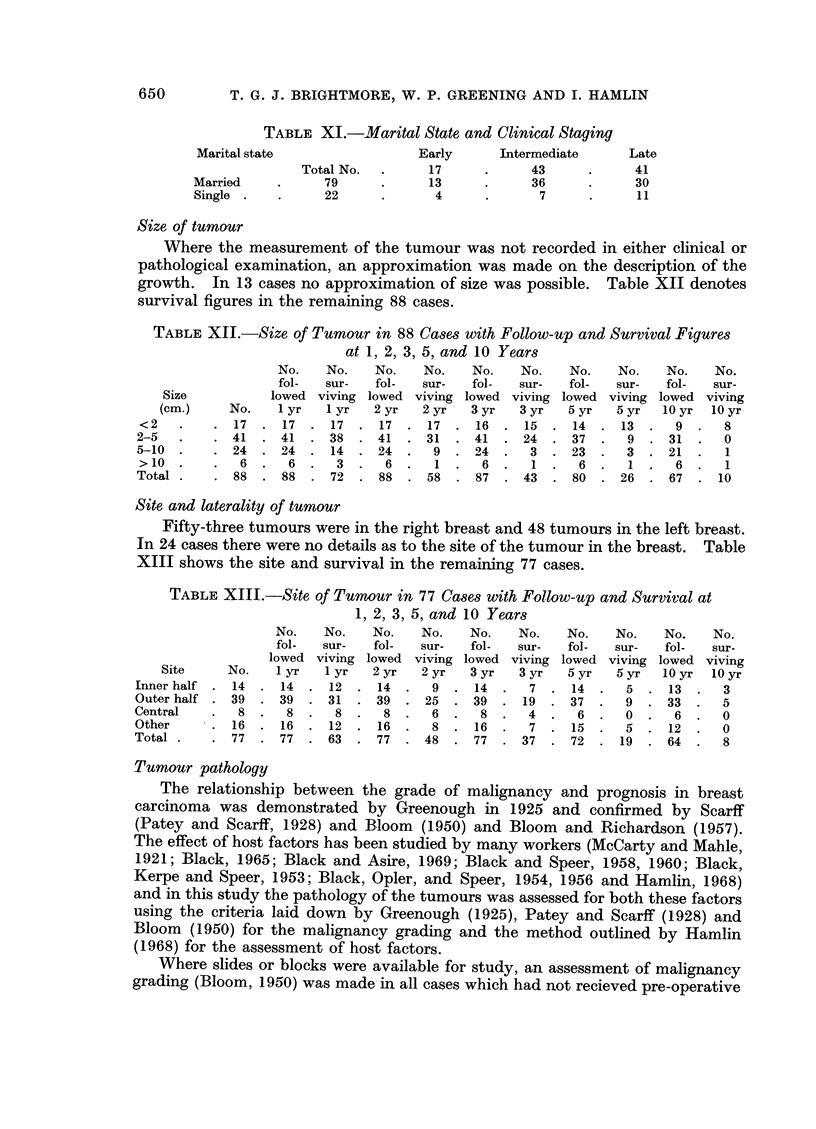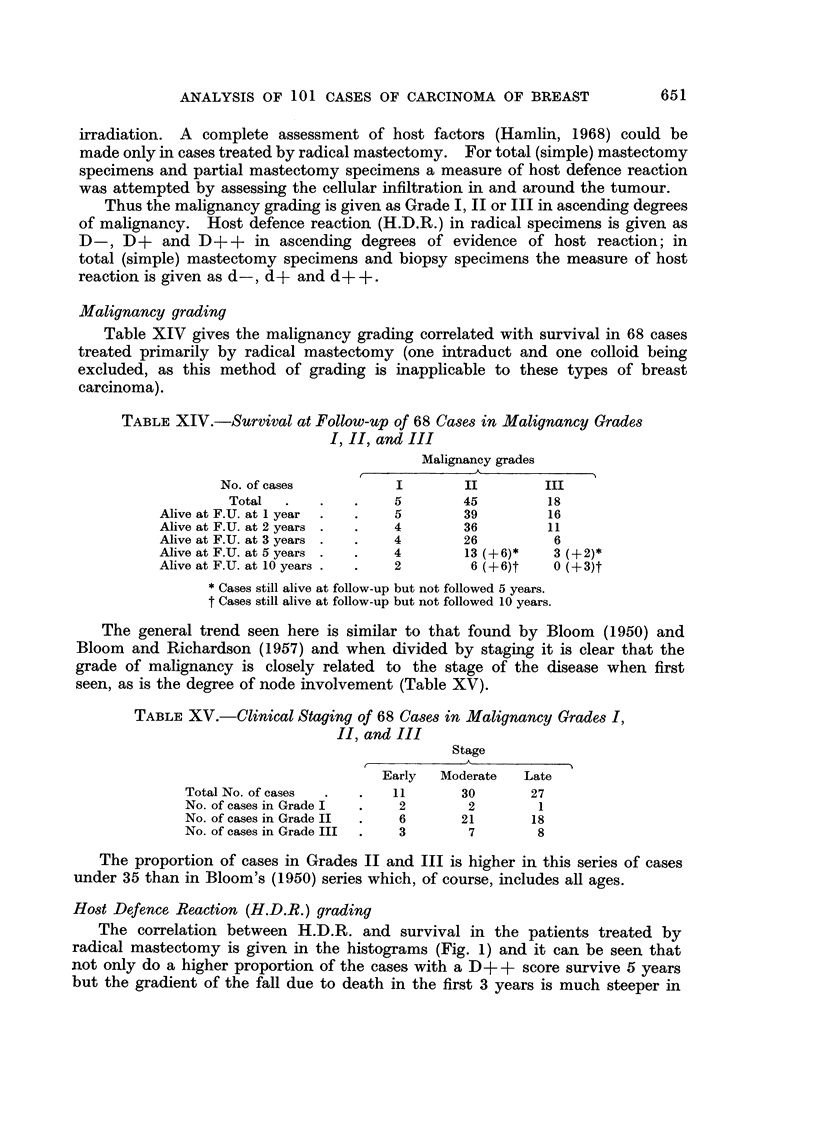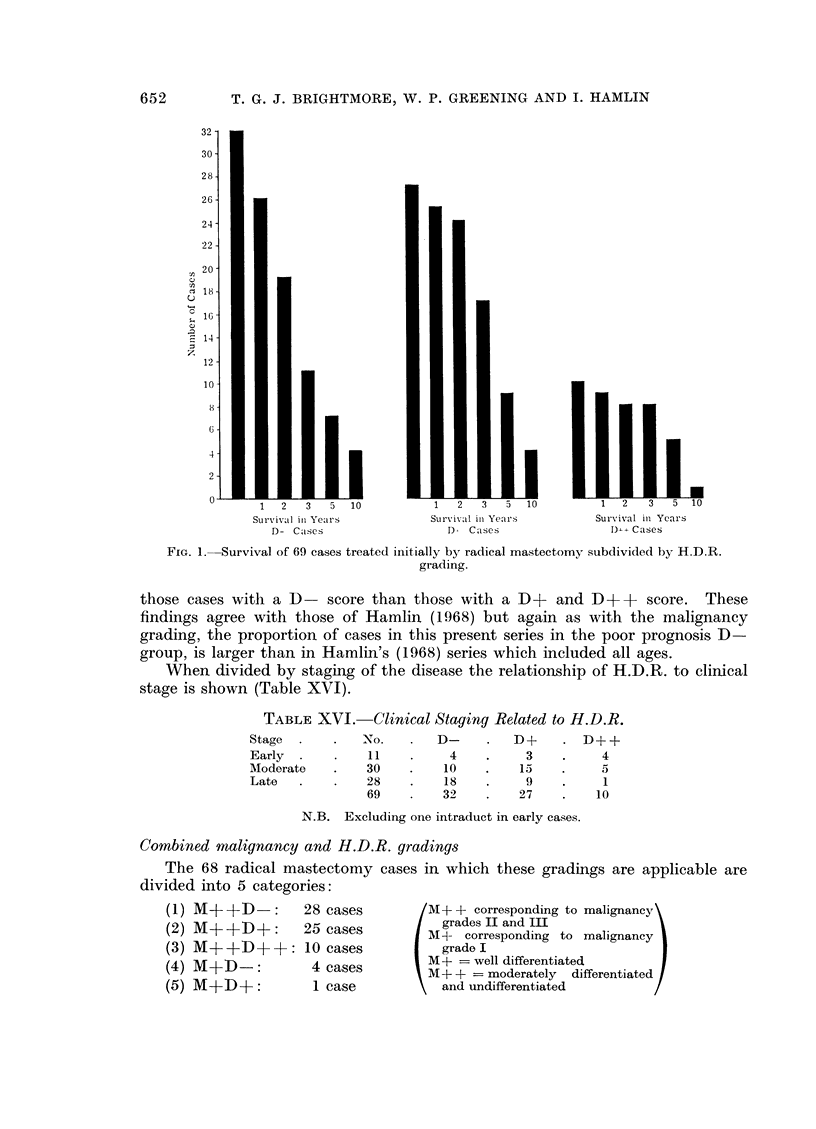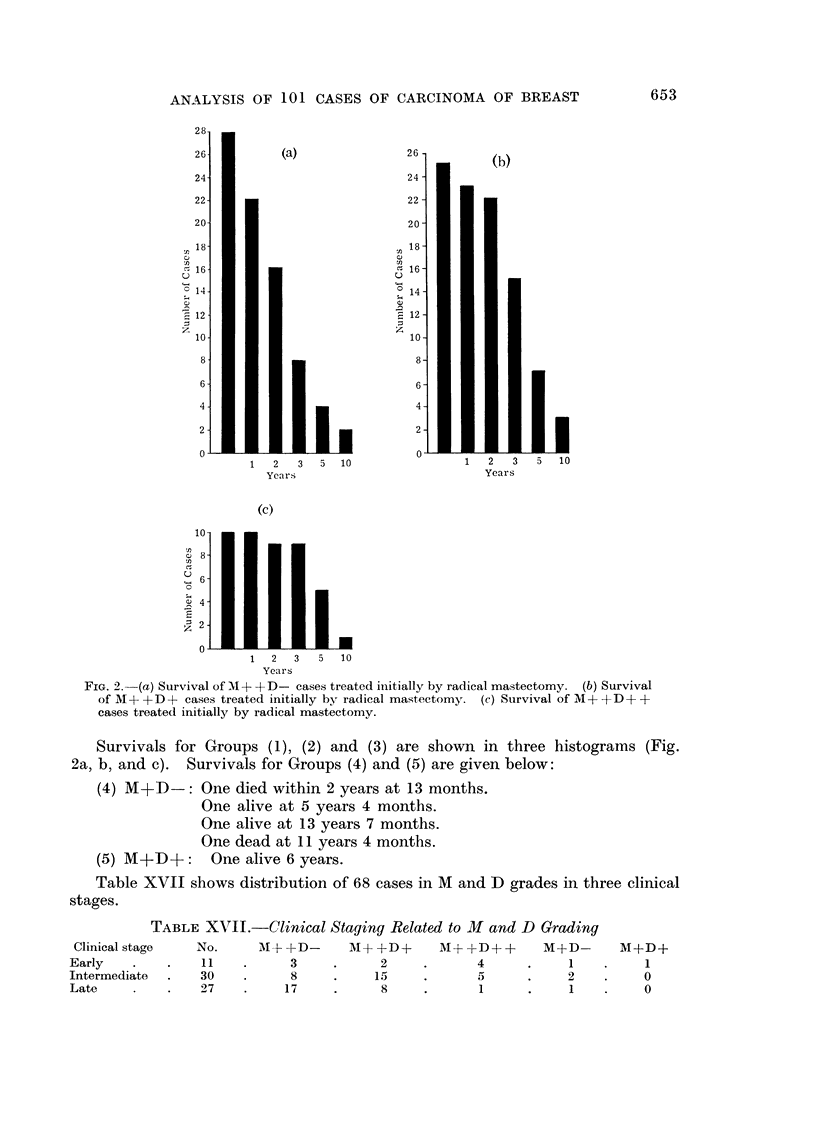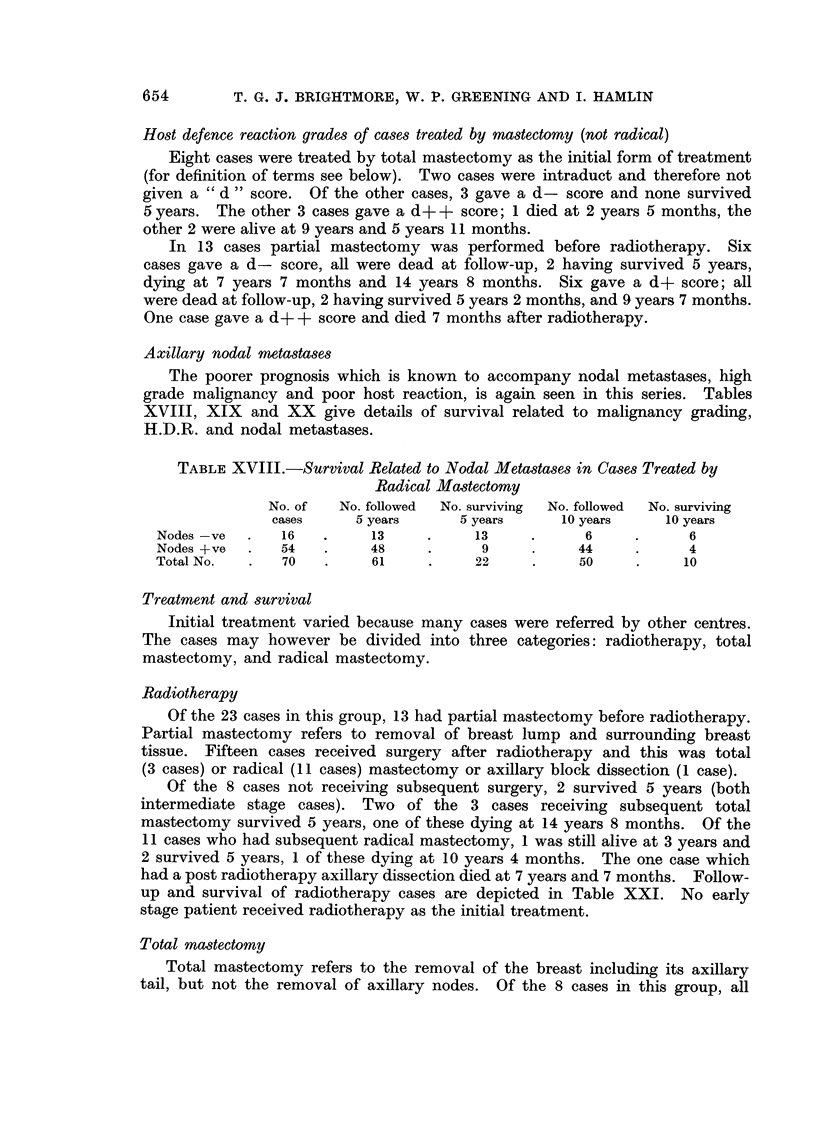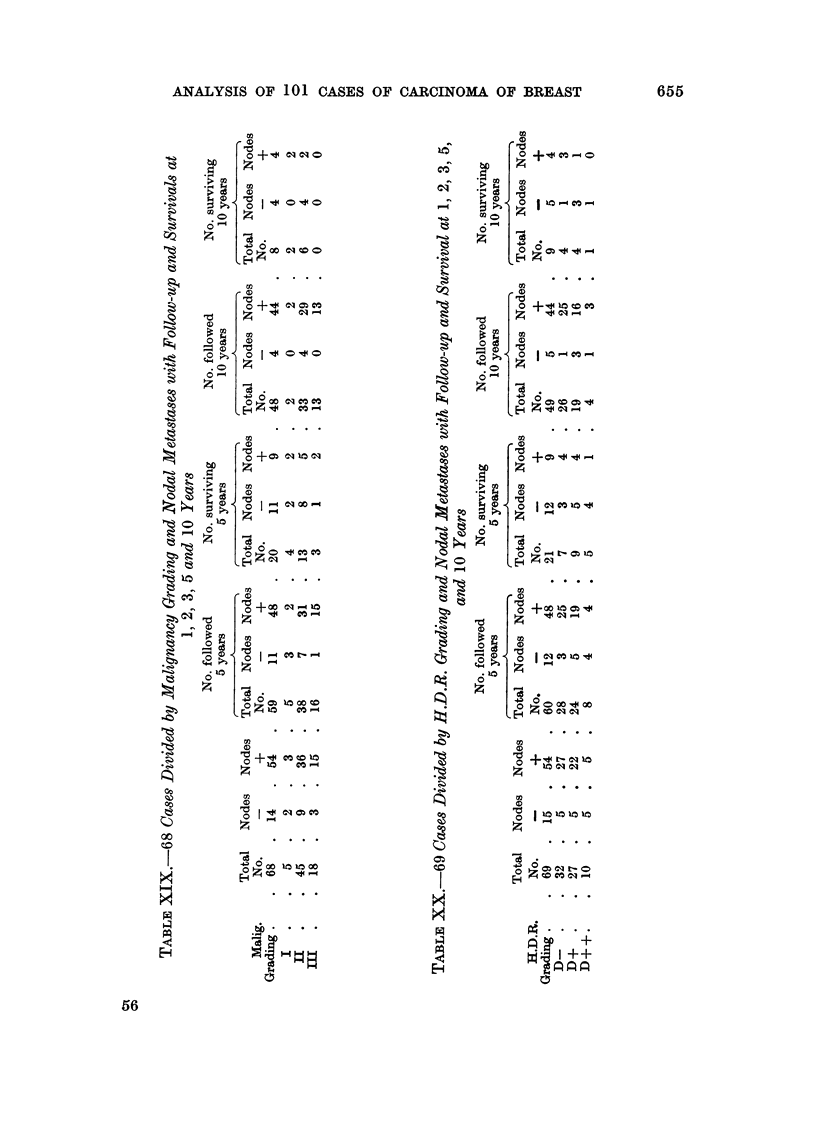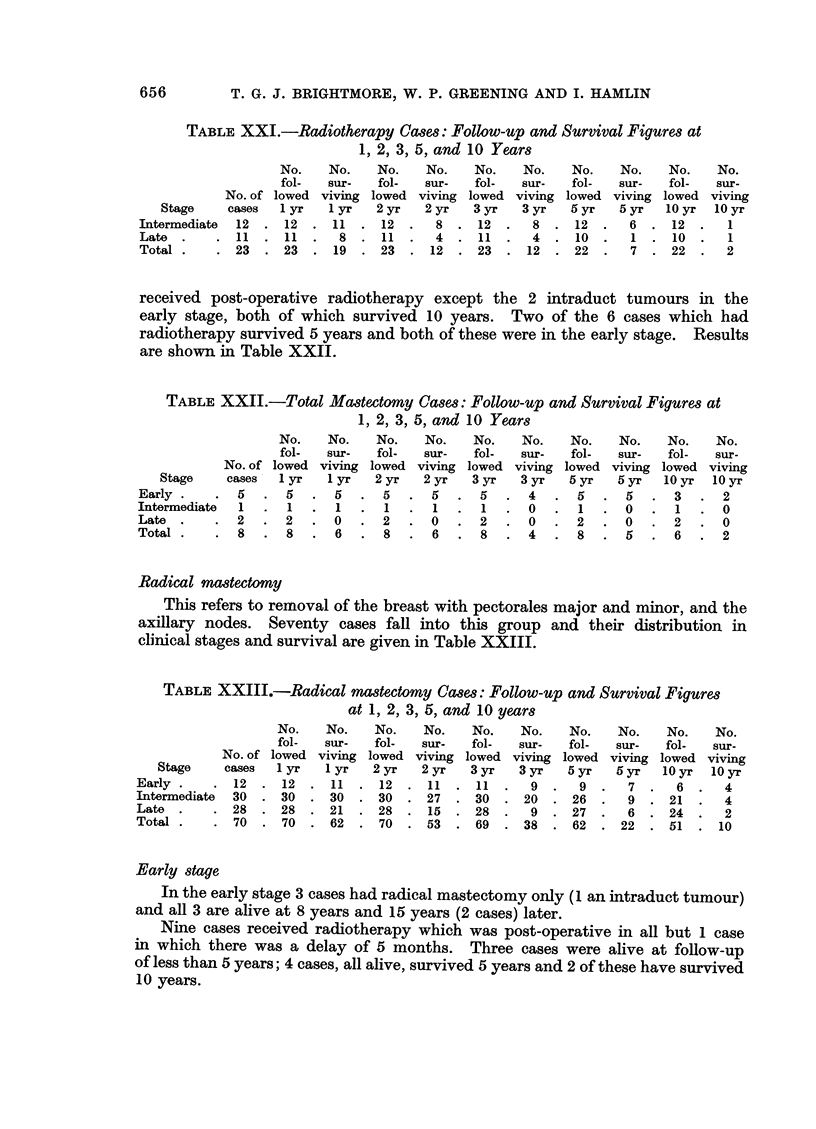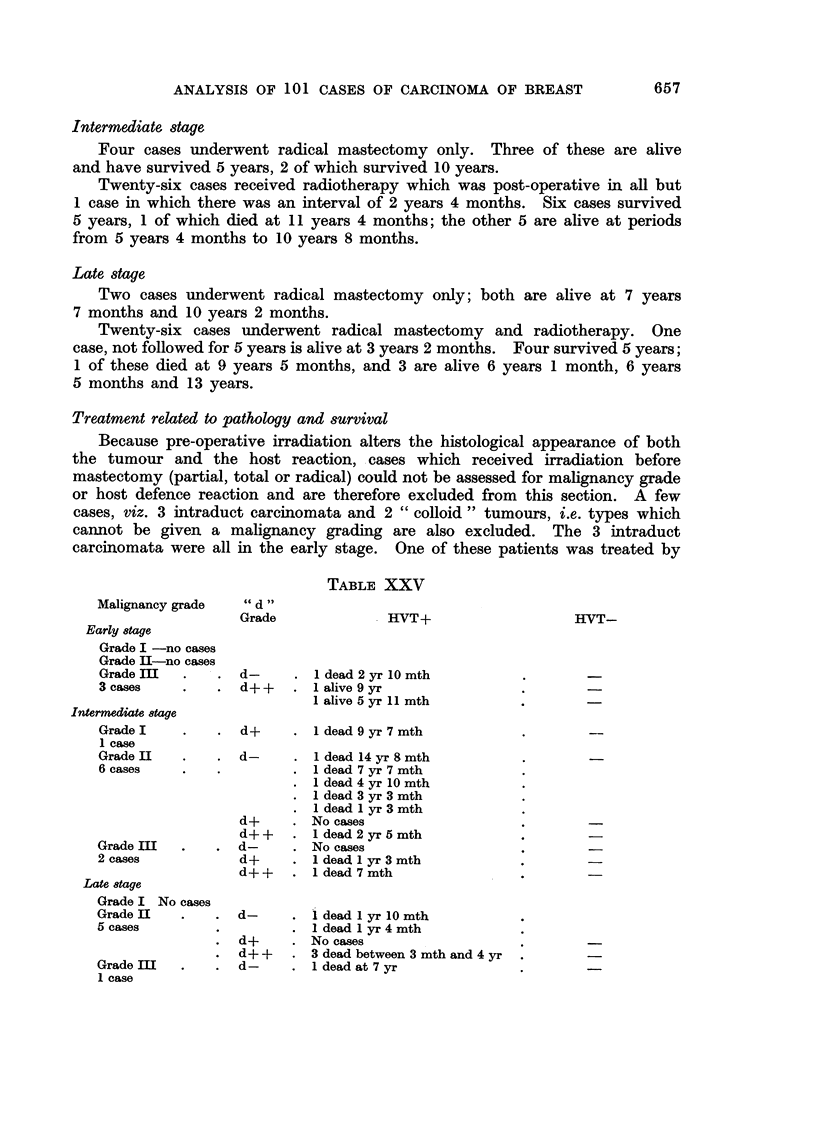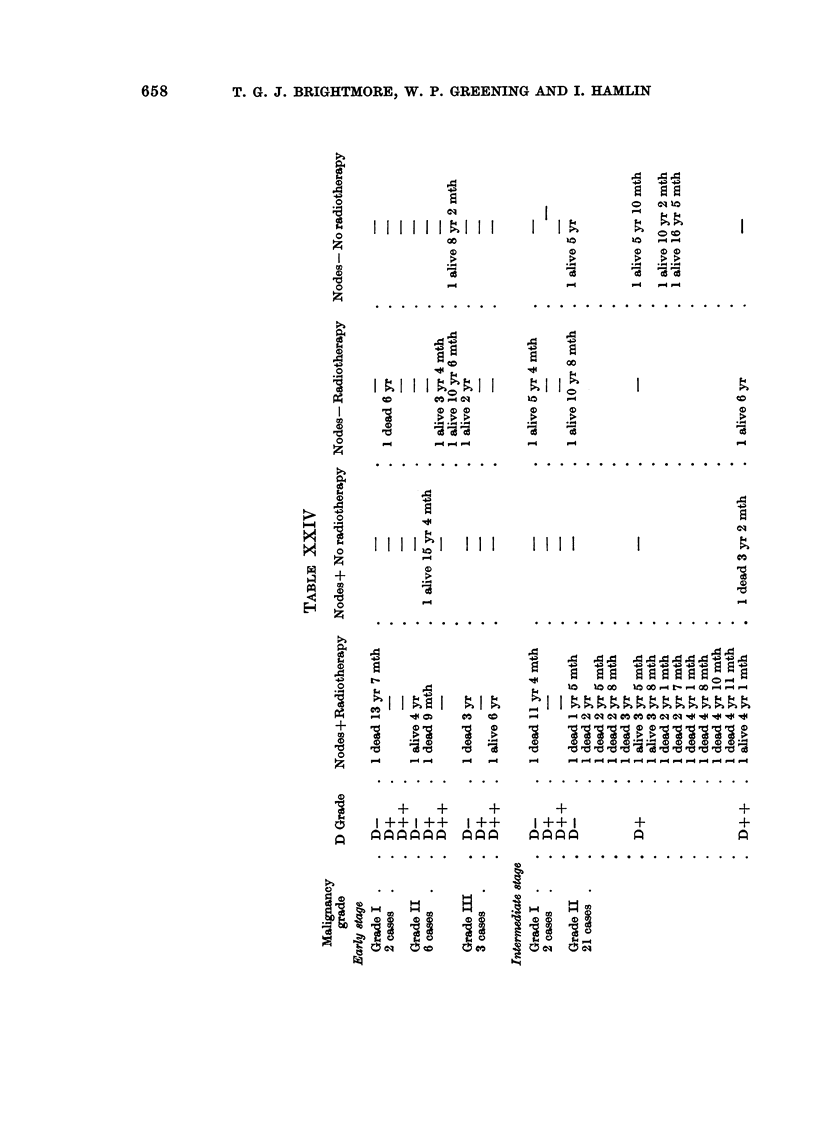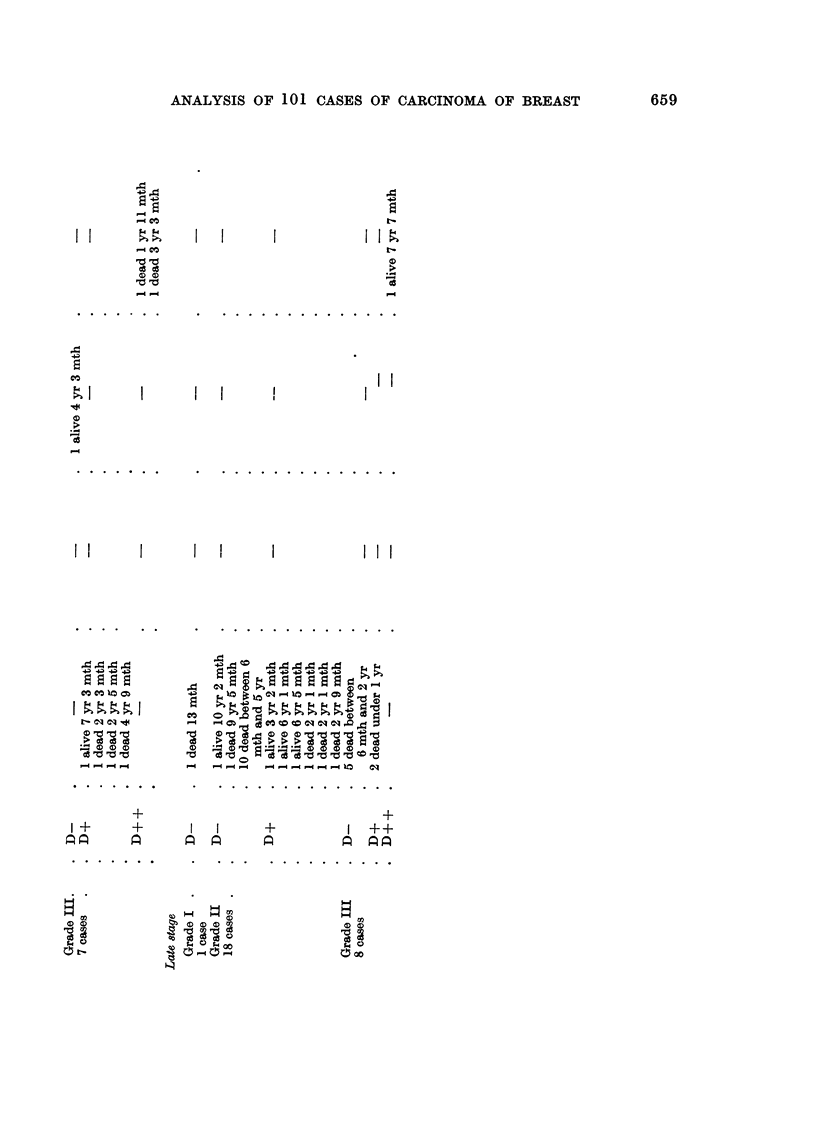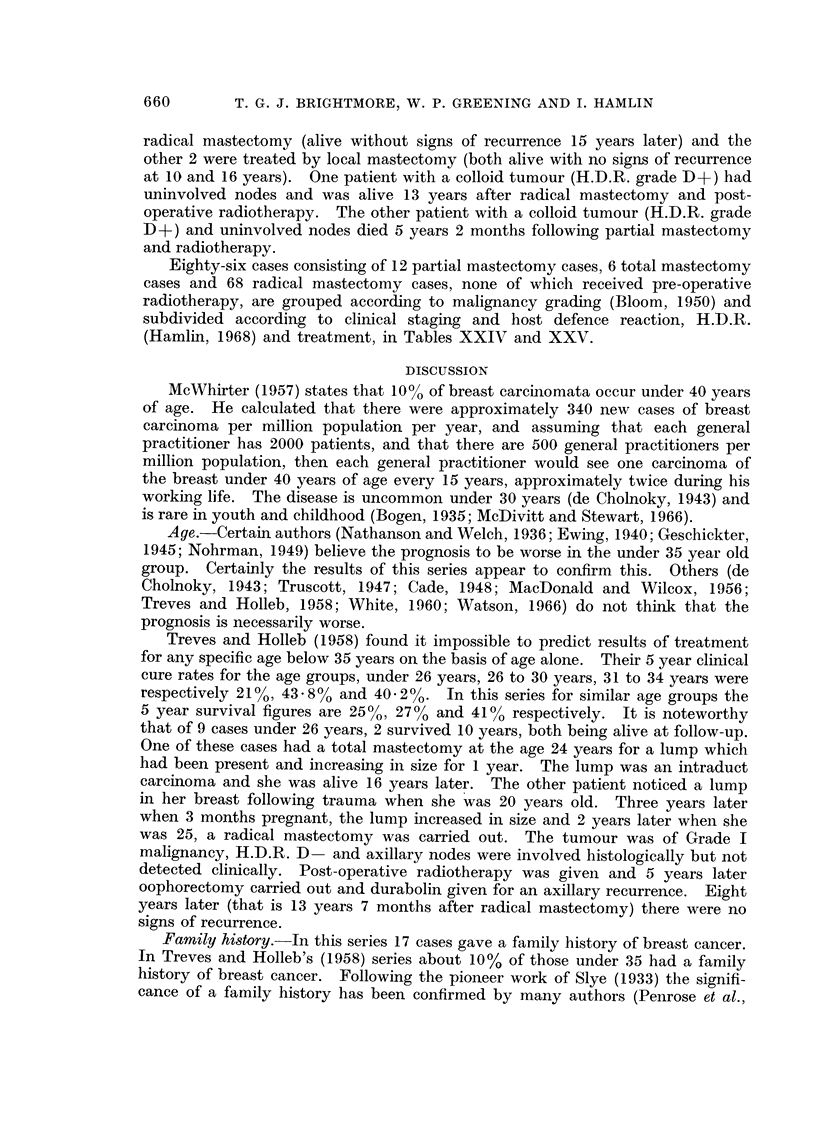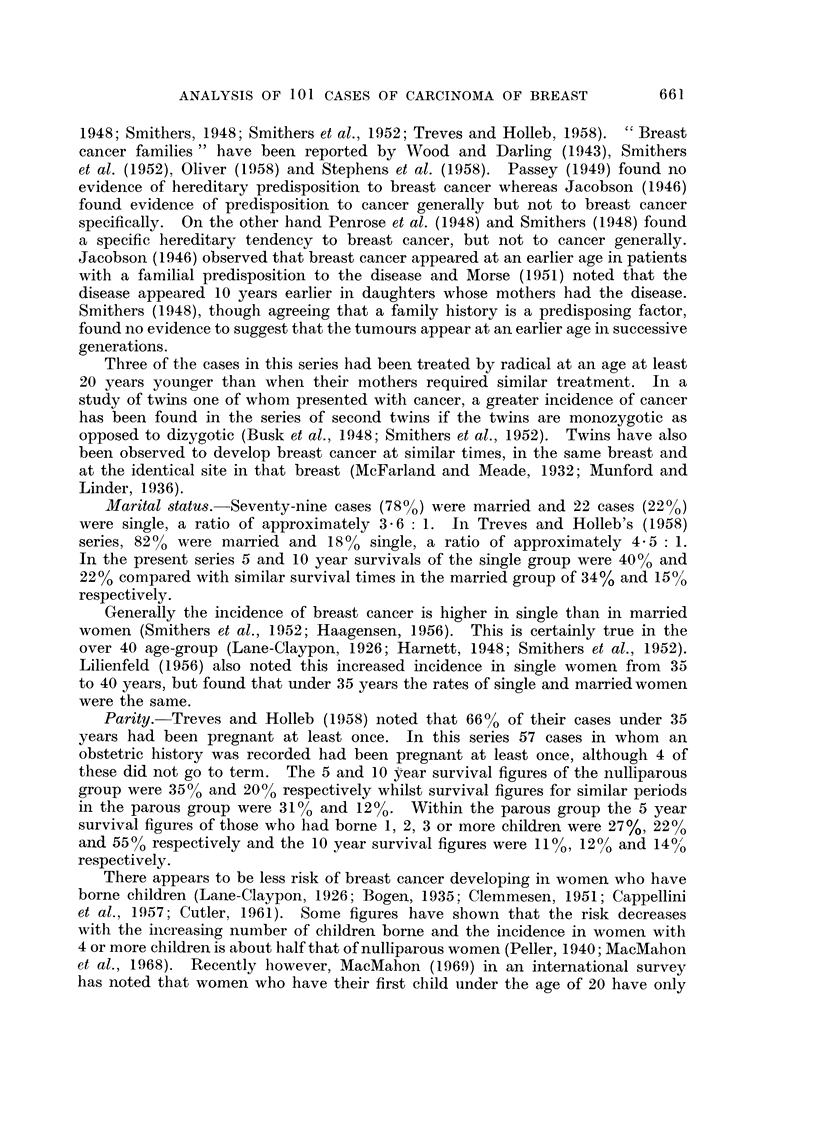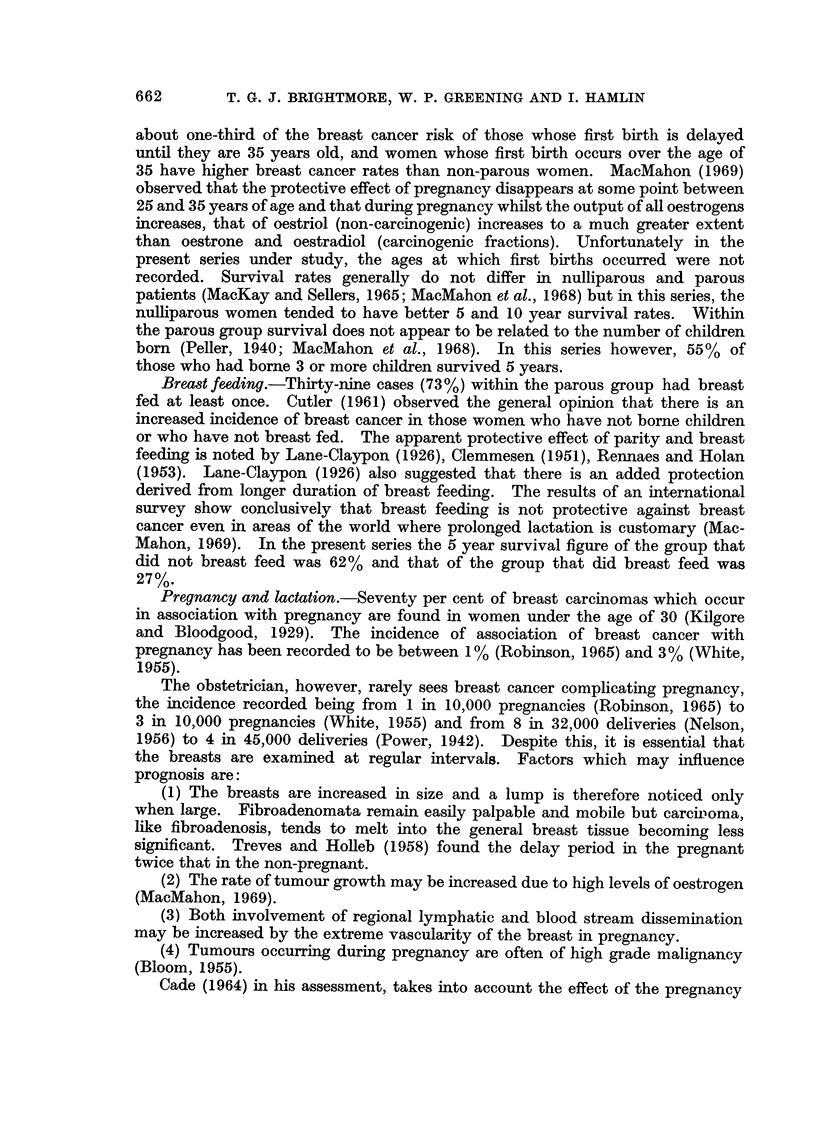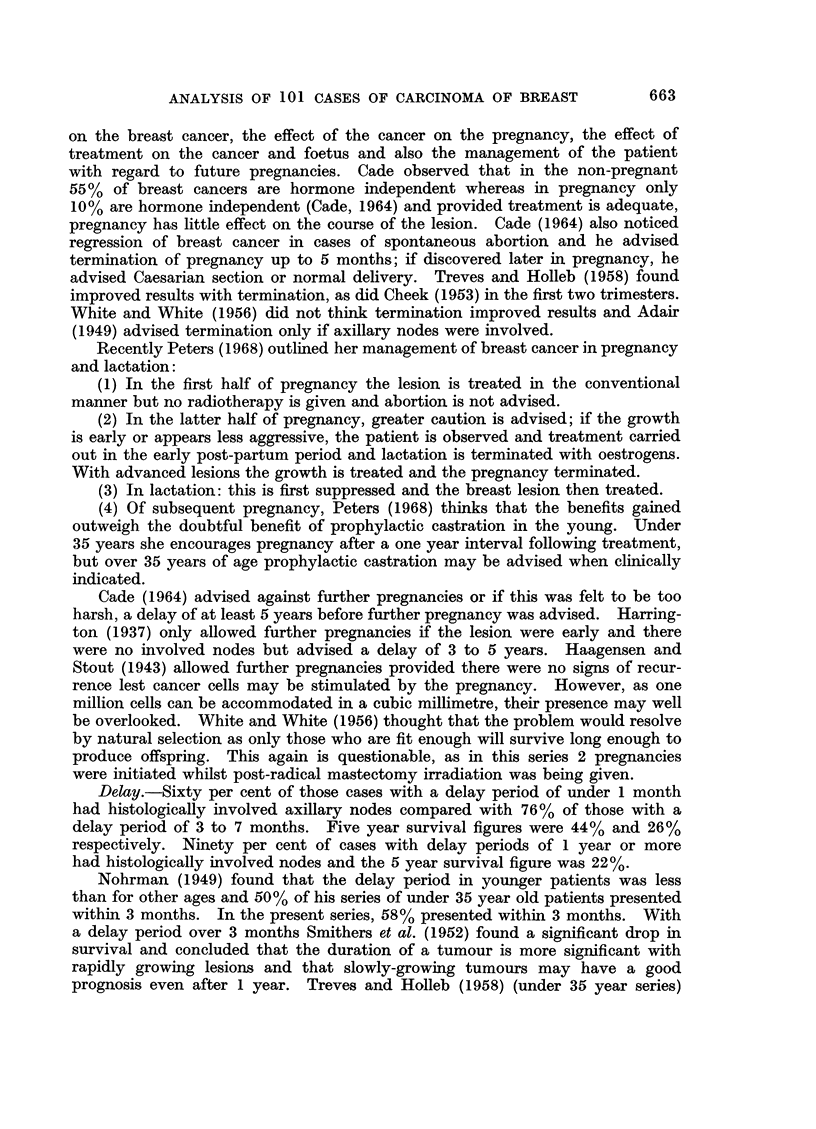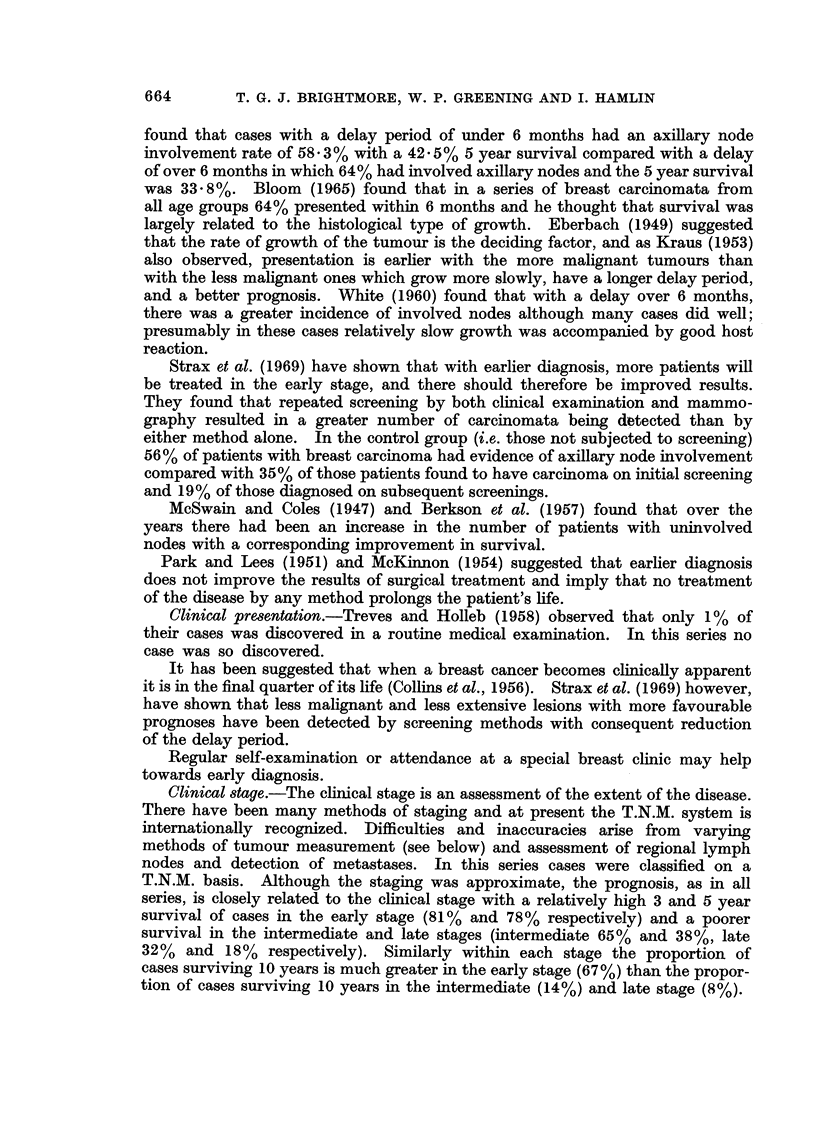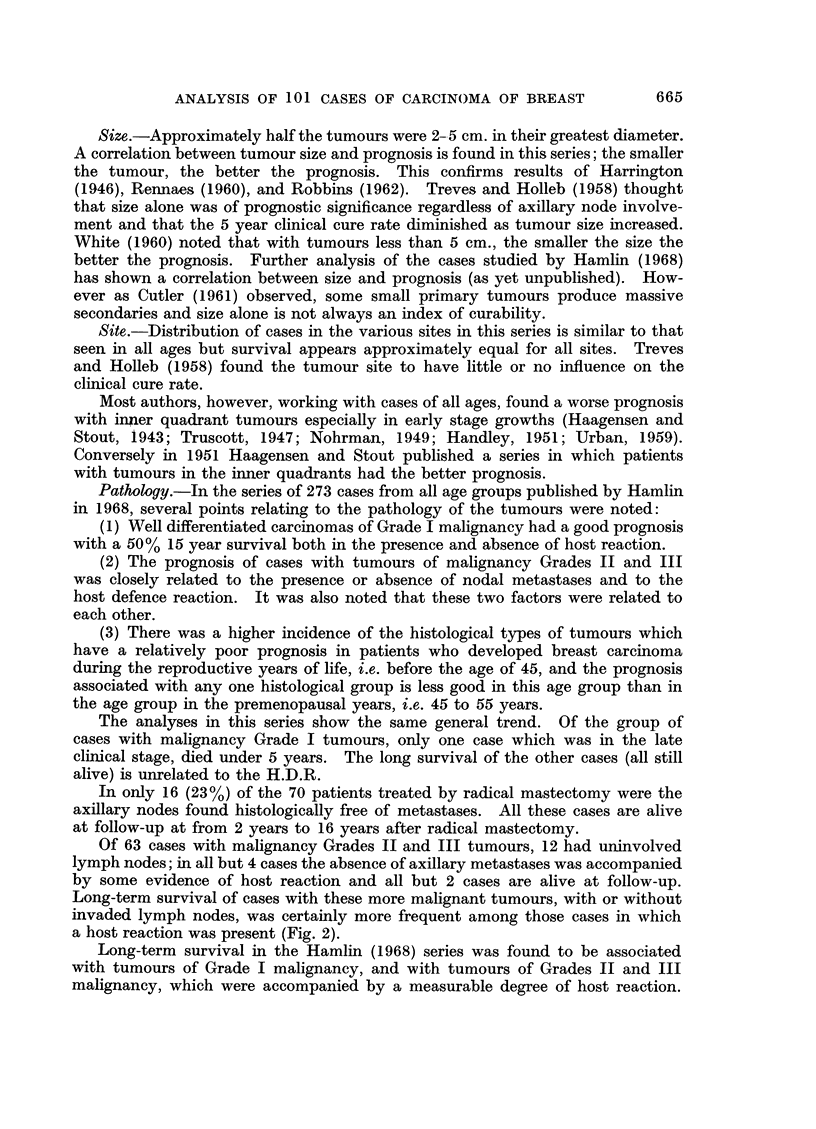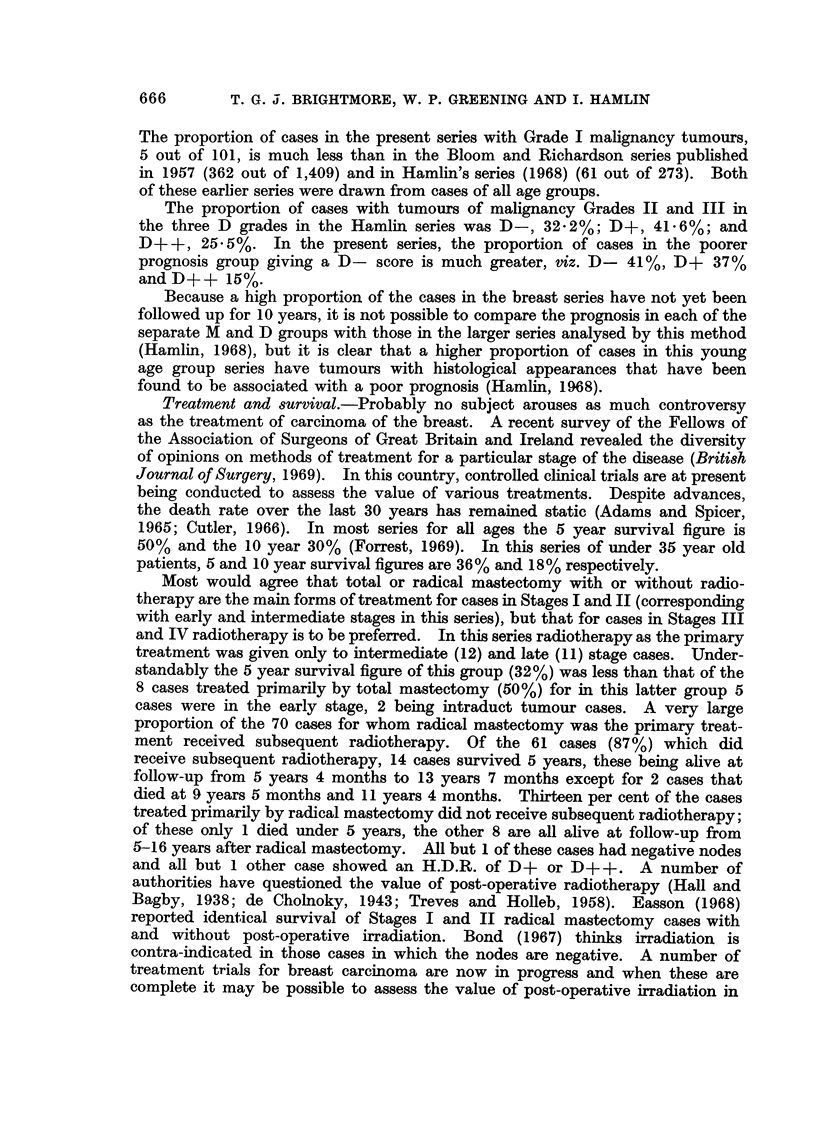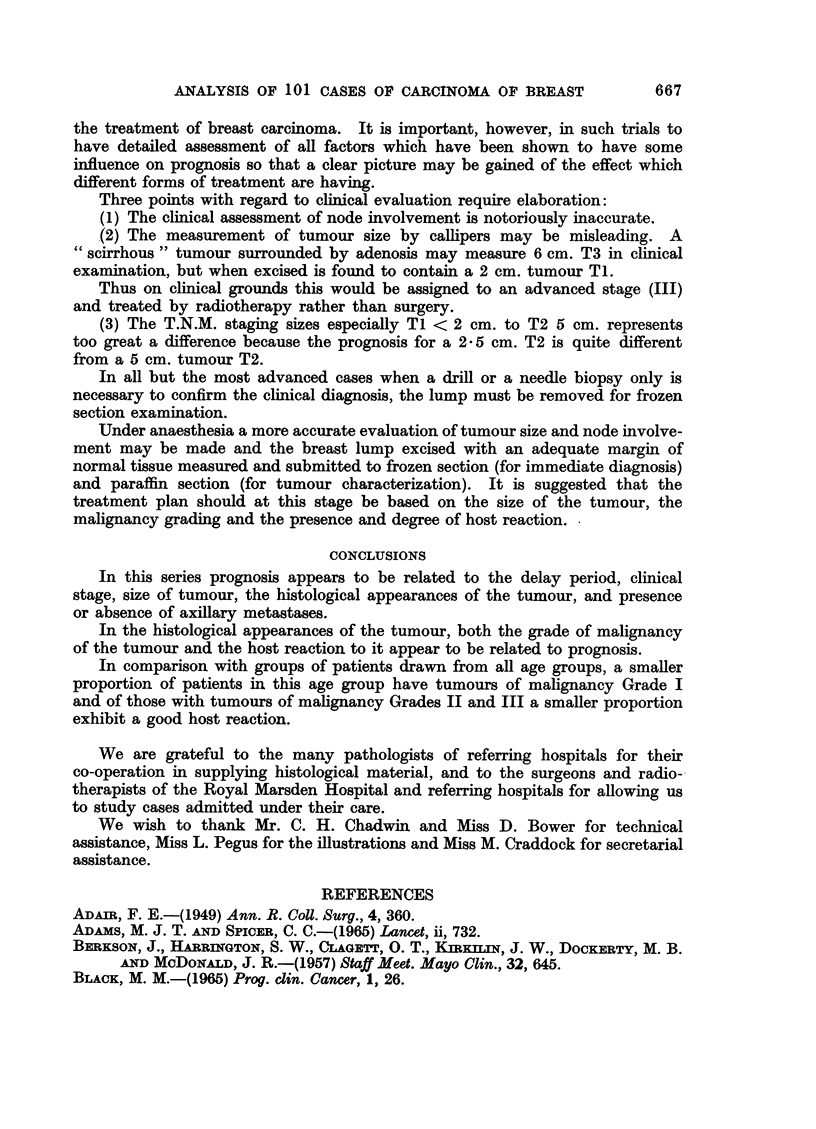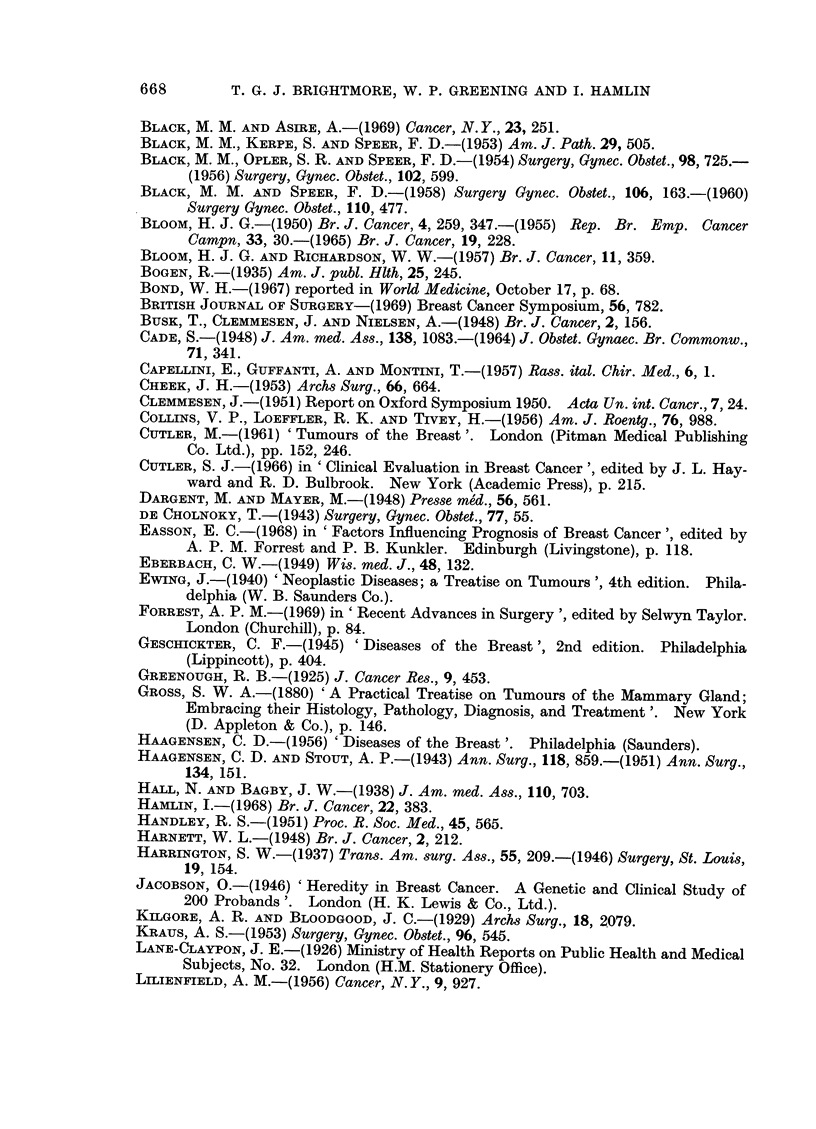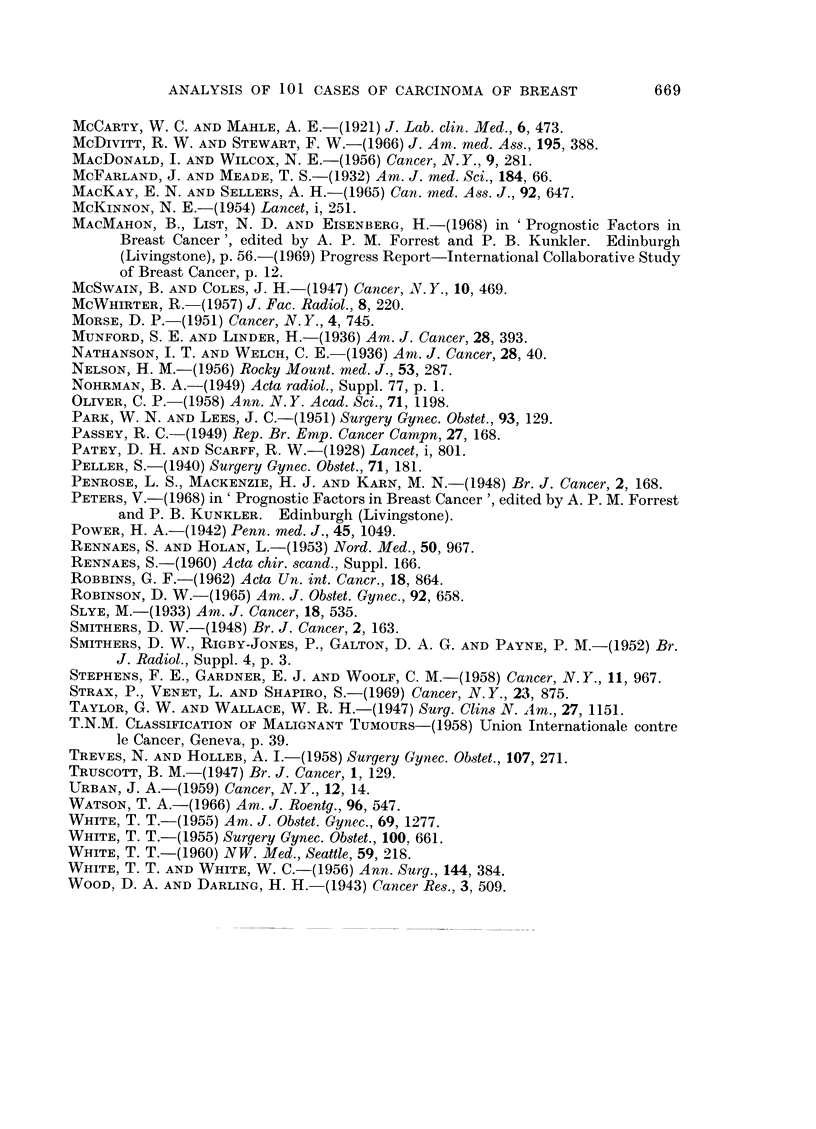# An Analysis of Clinical and Histopathological Features in 101 Cases of Carcinoma of Breast in Women Under 35 Years of Age

**DOI:** 10.1038/bjc.1970.78

**Published:** 1970-12

**Authors:** T. G. J. Brightmore, W. P. Greening, Iris Hamlin

## Abstract

An analysis of 101 cases of breast carcinoma occurring in patients under 35 years of age is presented with details of clinical stage, site, size and histological appearances of the tumour. Various factors are correlated with survival and the prognosis of the patient under 35 years is found to be closely related to the histological appearances of the tumour, which are reflected in the clinical stage at presentation. The question of treatment of carcinoma of the breast in the young woman is discussed.


### AN ANALYSIS OF CLINICAL AND HISTOPATHOLOGICAL FEATURES IN 101 CASES OF CARCINOMA OF BREAST IN WOMEN UNDER 35 YEARS OF AGE

T. G. J. BRIGHTMORE,* W. P. GREENING AND IRIS HAMLIN

*From the Breast Unit, Royal Marsden Hospital, London, S.W.3*

Received for publication June 25, 1970

**SUMMARY.—An analysis of 101 cases of breast carcinoma occurring in patients under 35 years of age is presented with details of clinical stage, site, size and histological appearances of the tumour. Various factors are correlated with survival and the prognosis of the patient under 35 years is found to be closely related to the histological appearances of the tumour, which are reflected in the clinical stage at presentation. The question of treatment of carcinoma of the breast in the young woman is discussed.**

THE treatment of breast cancer at any age or at any stage is a subject constantly under discussion. Opinions differ widely and categorical statements are made often without any foundation. It is unlikely that any agreement will be reached until much more is known about the aetiology of the disease.

At the present time therapy must be based on the knowledge available; this can be obtained by a study of the results of treatment retrospectively and it should be possible to give a reasoned opinion when confronted with a patient with breast cancer. Perhaps the two most difficult problems which face the clinician are the diagnosis of breast tumours in young patients and the decision as to the best plan of treatment. This is particularly important in patients under the age of 35 years who fall into a special category. They may be unmarried and therefore naturally opposed to the removal of a breast. They may be pregnant, when the problem of treatment is particularly difficult or they may have a family and have every reason to hope therefore for a long period of survival.

Carcinoma of the breast in the young is regarded by some authors as having a poor prognosis (Ewing, 1940; Taylor and Wallace, 1947) and others that it is no worse than in other age groups (de Cholnoky, 1943; Cade, 1948; Bloom, 1950; MacDonald and Wilcox, 1956). It was universally recognized that breast cancer during pregnancy or lactation had a poor prognosis and was for a long time considered to be inoperable (Gross, 1880). However, Harrington (1937) showed that the prognosis is not always as bad as it had been considered to be in the past, and Dargent and Mayer (1948) noted that in cases with uninvolved nodes the prognosis was certainly better than in cases uncomplicated by pregnancy or lactation. Smithers *et al.* (1952) reported 9 cases of breast cancer occurring concurrently with pregnancy and while 7 failed to survive more than 18 months, 2 remained alive and well, 1 without recurrence at 5 years, the other at 10 years.

While opinions differ fundamentally with regard to treatment, and although

* Present address: Westminster Hospital, London, S.W.1.

in many cases it is not of importance since the survival time is so short, nevertheless an attempt must be made to give the best possible treatment to those patients who are in fact going to survive for more than 5 years. A retrospective appraisal of patients under 35 years of age treated at the Royal Marsden Hospital over the last 20 years was undertaken in order to formulate some conclusions as to the best treatment for these patients.

### MATERIAL

One hundred and fifty-eight cases of breast carcinoma in women under 35 years of age were seen at the Royal Marsden Hospital in the years 1947 to 1966. One hundred and one of these cases have been retrospectively reviewed in clinical and pathological detail.

The cases were at various stages of their disease, some requiring initial treatment, and others evaluation following treatment elsewhere. As was inevitable with referrals from other centres, both in this country and abroad, clinical and pathological details were sometimes incomplete and where possible these were obtained from the referring centre. However, 54 cases had to be excluded because pathological material could not be obtained for reassessment. Three other cases were lost to follow-up within 1 to 3 years, and were excluded.

### RESULTS

### *Age and age group*

The age at which initial treatment was carried out is shown in Table I. Approximately 50% were under 32 years of age.

TABLE I.—*Distribution of Cases According to Age*

| | Age in years | | | | | | | | | | | |
|---|---|---|---|---|---|---|---|---|---|---|---|---|
| | 23 | 24 | 25 | 26 | 27 | 28 | 29 | 30 | 31 | 32 | 33 | 34 |
| Number of cases | 4 | 4 | 1 | 3 | 6 | 4 | 6 | 11 | 14 | 14 | 17 | 17 |

Table II denotes breakdown into age groups with 5 and 10 year survival figures.

TABLE II.—*Details of* 101 *Cases under* 35 *Years with Survivals of Age Groups of Cases Followed* 5 *and* 10 *Years*

| Number of cases | | Number followed 5 years | Number surviving 5 years | Number followed 10 years | Number surviving 10 years |
|---|---|---|---|---|---|
| Total | 101 | 92 | 33 | 79 | 14 |
| No. under 26 years | 9 | 8 | 2 | 8 | 2 |
| No. 26–30 years | 28 | 26 | 7 | 25 | 2 |
| No. 31–34 years | 64 | 58 | 24 | 46 | 10 |

### *Family history*

In many cases no details of family history were recorded or available but 17 of this series had near relations with breast cancer of whom 6 were the patient's mother, 7 were maternal relations, 2 were sisters and 2 were paternal relations.

Details of the ages of these relatives at presentation and their follow-up are inadequate for further study, but it is of interest that 2 patients in this series, aged 31 and 30, had mothers whose breast cancer was treated at 50 and 54

respectively. A 28 year old patient in this group had a mother and maternal grandmother treated when they were 54 and 45 respectively.

*Marital status*

Of the 101 cases, 79 were married and 22 single. There is little difference in survival in the two groups (Table III).

TABLE III.—*Details of Married and Single Cases with Follow-up and Survivals at* 1, 2, 3, 5, *and* 10 *Years*

| Marital state | Total | No. followed 1 year | No. surviving 1 year | No. followed 2 years | No. surviving 2 years | No. followed 3 years | No. surviving 3 years | No. followed 5 years | No. surviving 5 years | No. followed 10 years | No. surviving 10 years |
|---|---|---|---|---|---|---|---|---|---|---|---|
| Married | 79 | 79 | 67 | 79 | 55 | 78 | 40 | 72 | 25 | 61 | 10 |
| Single | 22 | 22 | 20 | 22 | 16 | 22 | 14 | 20 | 8 | 18 | 4 |

*Parity*

An obstetric history was not recorded in 10 cases. Thirty-four patients had never been pregnant and of the remaining 57, 4 had miscarried in the first 3 months and are regarded as being nulliparous in the survival figures of Table IV.

TABLE IV.—*Details of Nulliparous and Parous Cases with Follow-up and Survivals at* 1, 2, 3, 5, *and* 10 *Years*

| | Total | No. followed 1 year | No. surviving 1 year | No. followed 2 years | No. surviving 2 years | No. followed 3 years | No. surviving 3 years | No. followed 5 years | No. surviving 5 years | No. followed 10 years | No. surviving 10 years |
|---|---|---|---|---|---|---|---|---|---|---|---|
| Nulliparous | 38 | 38 | 35 | 38 | 31 | 37 | 24 | 34 | 12 | 30 | 6 |
| Parous | 53 | 53 | 42 | 53 | 30 | 53 | 23 | 49 | 15 | 41 | 5 |

Table V compares survival in parous groups.

TABLE V.—*Details of Parous Cases with Follow-up and Survivals at* 1, 2, 3, 5, *and* 10 *Years*

| No. of children | No. of cases | No. followed 1 year | No. surviving 1 year | No. followed 2 years | No. surviving 2 years | No. followed 3 years | No. surviving 3 years | No. followed 5 years | No. surviving 5 years | No. followed 10 years | No. surviving 10 years |
|---|---|---|---|---|---|---|---|---|---|---|---|
| 1 | 23 | 23 | 19 | 23 | 13 | 23 | 11 | 22 | 6 | 18 | 2 |
| 2 | 20 | 20 | 15 | 20 | 10 | 20 | 5 | 18 | 4 | 16 | 2 |
| 3 and over | 10 | 10 | 8 | 10 | 7 | 10 | 7 | 9 | 5 | 7 | 1 |
| Total | 53 | 53 | 42 | 53 | 30 | 53 | 23 | 49 | 15 | 41 | 5 |

TABLE VI.—*Details of Breast Feeding and Non-breast Feeding Cases with Follow-up and Survivals at* 1, 2, 3, 5, *and* 10 *Years*

| | Total | No. followed 1 year | No. surviving 1 year | No. followed 2 years | No. surviving 2 years | No. folowed 3 years | No. surviving 3 years | No. followed 5 years | No. surviving 5 years | No. followed 10 years | No. surviving 10 years |
|---|---|---|---|---|---|---|---|---|---|---|---|
| Breast feeding | 39 | 39 | 32 | 39 | 21 | 39 | 16 | 37 | 10 | 33 | 6 |
| Non-breast feeding | 9 | 9 | 7 | 9 | 7 | 9 | 6 | 8 | 5 | 5 | 0 |

*Breast feeding*

Of the 55 parous cases, 39 had breast fed at least once, although details of duration of feeding were unrecorded. Nine cases had not breast fed and for the remaining 7 cases no details were recorded.

TABLE VII.—*Details of* 17 *Patients with Breast Carcinoma Associated with Pregnancy or Lactation*

| | Delay | Stage | Treatment | Prognosis |
|---|---|---|---|---|
| Before pregnancy | 1 yr | Late | Radiotherapy at 20 weeks of pregnancy followed by radical mastectomy | N.S.R. 3 yr 4 mth |
| | 5 mth | Late | Partial mastectomy and radiotherapy at 5 mth and hysterectomy | Dead 3 mth |
| During Pregnancy | 4 mth | Early | Total mastectomy only at 24 wk of pregnancy | N.S.R. 5 yr 11 mth |
| | 1 yr | Late | 9 mth post-partum partial mastectomy and radiotherapy | Dead 1 yr 3 mth |
| 1st Trimester | 5 mth | moderate | At 5 mth of pregnancy partial mastectomy, radiotherapy and total mastectomy | Dead 5 yr 3 mth |
| | 2 yr | Early | 17 mth after delivery radical mastectomy and radiotherapy | Alive 13 yr 7 mth |
| 2nd Trimester | 3 mth | Early | Radical mastectomy at 6 mth of pregnancy and after delivery radiotherapy | Alive 4 yr |
| 3rd Trimester | 10 mth | Late | 9 mth after delivery, radiotherapy and radical mastectomy | Dead 1 yr 1 mth |
| | 6 mth | Late | 6 mth after delivery radical mastectomy and radiotherapy | Dead 11 mth |
| Post-partum and lactation | 6 wk | Late | 15 wk after delivery, radical mastectomy and radiotherapy | Dead 1 yr 2 mth |
| | 14 mth | Late | 14 mth after delivery radical mastectomy and radiotherapy | Dead 1 mth |
| | < 1 mth | Early | 4 mth after delivery, radical mastectomy and radiotherapy | Dead 2 yr 2 mth |
| | 6 mth | Late | 10 mth after delivery, radical mastectomy and radiotherapy | Dead 6 mth |
| Pregnancy following treatment | < 1 mth | Early | Pregnancy 4 yr after radical mastectomy | N.S.R. 15 yr 4 mth |
| | 2 yr | Moderate | Pregnancy 2½ yr after radical mastectomy and radiotherapy—*terminated* | Alive 3 yr 8 mth |
| | 5 mth | Late | Pregnancy 2 mth after radical mastectomy whilst having radiotherapy—*terminated* | Dead 2 yr 11 mth |
| | 6 mth | Late | Pregnancy 2 mth following radical mastectomy whilst receiving radiotherapy. Miscarriage at 10 wk, within 3 wk pregnant again and *terminated* at 6 wk | Alive 6 yr 5 mth |

*Pregnancy and lactation*

Seventeen cases in this category were divided into groups depending on whether the lump was noticed before, during or soon after pregnancy, or whether the patient had become pregnant following treatment. The results are shown in Table VII. Delay in this table refers to the period between the discovery of the lump or first symptom and the institution of treatment. For an explanation of stage see below under Clinical Stage.

Both cases with lumps found before pregnancy were in the late clinical stage and both had involved nodes.

Of the 7 cases in which lumps were discovered during pregnancy all were allowed to term, 2 receiving initial treatment whilst pregnant (radical mastectomy followed by post-partum radiotherapy); 3 cases survived 5 years. One of these cases, despite a delay of nearly 20 months, had no histological evidence of axillary metastases and is alive 13 years and 7 months later.

In 4 cases a lump was discovered during lactation; all had involved nodes and were dead within 2 years 2 months.

Four cases became pregnant following treatment. Two of these were terminated whilst receiving post-radical mastectomy radiotherapy, one dying within 3 years, the other alive 6 years and 5 months later. The other 2 who became pregnant $2\frac{1}{2}$ years and 4 years after treatment (radical mastectomy with post-operative radiotherapy, and radical mastectomy only, respectively) were allowed to go to term and are alive 3 years 8 months and 15 years 4 months later respectively.

*Delay*

The period of delay between the discovery of a lump by the patient (or the first symptom) and the start of treatment was recorded in 99 cases and is tabulated (Table VIII).

TABLE VIII.—*Delay Before Treatment in 99 Cases*

| Delay in months | Less than 1 month | 1 | 2 | 3 | 4 | 5 | 6 | 7 | 8 | 9 | 10 | 11 | 12 months and over |
|---|---|---|---|---|---|---|---|---|---|---|---|---|---|
| No. of cases | 25 | 15 | 18 | 10 | 2 | 5 | 6 | 2 | 1 | 1 | 1 | 2 | 11 |

In the 25 cases with a delay period of less than one month, 15 cases had histological evidence of axillary node metastases. Twenty-five patients had a delay period of 3–7 months and 19 of these cases had histologically involved nodes. Of 11 patients with a delay of 1 year or more, 9 had histologically involved nodes.

Survival figures of the three groups are shown in Table IX.

TABLE IX.—*Details of Delay Periods with Follow-up and Survival at 1, 2, 3, 5, and 10 Years*

| Delay (months) | No. of cases | No. followed 1 yr | No. surviving 1 yr | No. followed 2 yr | No. surviving 2 yr | No. followed 3 yr | No. surviving 3 yr | No. followed 5 yr | No. surviving 5 yr | No. followed 10 yr | No. surviving 10 yr |
|---|---|---|---|---|---|---|---|---|---|---|---|
| < 1 | 25 | 25 | 21 | 25 | 20 | 25 | 15 | 23 | 10 | 16 | 2 |
| 3–7 | 25 | 25 | 20 | 25 | 16 | 25 | 14 | 23 | 6 | 22 | 2 |
| 12 and over | 11 | 11 | 10 | 11 | 7 | 11 | 4 | 9 | 2 | 8 | 1 |

*Clinical presentation*

Ninety-five patients felt a lump in the breast (4 lumps being tender), 3 felt a lump in the axilla and 3 presented with nipple discharge (one serosanguinous, 2 serous). In no case was the lump discovered at a routine medical examination. The clinical diagnosis at hospital level was:

| | |
|---|---|
| (a) Carcinoma | 92 cases |
| (b) Fibro-cystic disease | 5 cases |
| (c) Duct papilloma | 2 cases |
| (d) Duct carcinoma | 1 case |
| (e) Fibroadenoma | 1 case |

*Clinical stage*

Accurate clinical staging was not possible in all cases, but an attempt has been made to regroup the cases according to the T.N.M. criteria of the U.I.C.C. classification. Where appropriate clinical information was not available this was deduced where possible from pathological information. This was often the case in respect of tumour size and therefore T1 or T2 NO MO group (Stage I or " early ") only includes tumours of 5 cm. or less. Some of these may have been recorded clinically as T3. Because the information about the size of the tumour and the state of the axillary nodes was not uniformly clinical or pathological, this grouping is largely artificial and not strictly comparable with clinical T.N.M. observations.

Cases have therefore been grouped as follows:

| | |
|---|---|
| Stage I (early): | T1 NO MO |
| | T2 NO MO |
| Stage II (intermediate): | T1 N1 MO |
| | T2 N1 MO |
| Stage III (late): | T1 N2 or N3 MO |
| | T2 N2 or N3 MO |
| | T3 NO, N1, N2 or N3 MO |
| | T4 NO, N1, N2 or N3, MO |

See Table X for staging and survival figures.

TABLE X.—*Clinical Staging of* 101 *Cases with Follow-up and Survivals at* 1, 2, 3, 5, *and* 10 *Years*

| Stage | No. of cases | No. followed 1 yr | No. surviving 1 yr | No. followed 2 yr | No. surviving 2 yr | No. followed 3 yr | No. surviving 3 yr | No. followed 5 yr | No. surviving 5 yr | No. followed 10 yr | No. surviving 10 yr |
|---|---|---|---|---|---|---|---|---|---|---|---|
| Early | 17 | 17 | 16 | 17 | 16 | 16 | 13 | 14 | 11 | 9 | 6 |
| Intermediate | 43 | 43 | 42 | 43 | 36 | 43 | 28 | 39 | 15 | 34 | 5 |
| Late | 41 | 41 | 29 | 41 | 19 | 41 | 13 | 39 | 7 | 36 | 3 |
| Total | 101 | 101 | 87 | 101 | 71 | 100 | 54 | 92 | 33 | 79 | 14 |

Table XI shows the clinical staging of married and single patients.

TABLE XI.—*Marital State and Clinical Staging*

| Marital state | Total No. | Early | Intermediate | Late |
|---|---|---|---|---|
| | | 17 | 43 | 41 |
| Married | 79 | 13 | 36 | 30 |
| Single | 22 | 4 | 7 | 11 |

*Size of tumour*

Where the measurement of the tumour was not recorded in either clinical or pathological examination, an approximation was made on the description of the growth. In 13 cases no approximation of size was possible. Table XII denotes survival figures in the remaining 88 cases.

TABLE XII.—*Size of Tumour in* 88 *Cases with Follow-up and Survival Figures at* 1, 2, 3, 5, *and* 10 *Years*

| Size (cm.) | No. | No. followed 1 yr | No. surviving 1 yr | No. followed 2 yr | No. surviving 2 yr | No. followed 3 yr | No. surviving 3 yr | No. followed 5 yr | No. surviving 5 yr | No. followed 10 yr | No. surviving 10 yr |
|---|---|---|---|---|---|---|---|---|---|---|---|
| <2 | 17 | 17 | 17 | 17 | 17 | 16 | 15 | 14 | 13 | 9 | 8 |
| 2–5 | 41 | 41 | 38 | 41 | 31 | 41 | 24 | 37 | 9 | 31 | 0 |
| 5–10 | 24 | 24 | 14 | 24 | 9 | 24 | 3 | 23 | 3 | 21 | 1 |
| >10 | 6 | 6 | 3 | 6 | 1 | 6 | 1 | 6 | 1 | 6 | 1 |
| Total | 88 | 88 | 72 | 88 | 58 | 87 | 43 | 80 | 26 | 67 | 10 |

*Site and laterality of tumour*

Fifty-three tumours were in the right breast and 48 tumours in the left breast. In 24 cases there were no details as to the site of the tumour in the breast. Table XIII shows the site and survival in the remaining 77 cases.

TABLE XIII.—*Site of Tumour in* 77 *Cases with Follow-up and Survival at* 1, 2, 3, 5, *and* 10 *Years*

| Site | No. | No. followed 1 yr | No. surviving 1 yr | No. followed 2 yr | No. surviving 2 yr | No. followed 3 yr | No. surviving 3 yr | No. followed 5 yr | No. surviving 5 yr | No. followed 10 yr | No. surviving 10 yr |
|---|---|---|---|---|---|---|---|---|---|---|---|
| Inner half | 14 | 14 | 12 | 14 | 9 | 14 | 7 | 14 | 5 | 13 | 3 |
| Outer half | 39 | 39 | 31 | 39 | 25 | 39 | 19 | 37 | 9 | 33 | 5 |
| Central | 8 | 8 | 8 | 8 | 6 | 8 | 4 | 6 | 0 | 6 | 0 |
| Other | 16 | 16 | 12 | 16 | 8 | 16 | 7 | 15 | 5 | 12 | 0 |
| Total | 77 | 77 | 63 | 77 | 48 | 77 | 37 | 72 | 19 | 64 | 8 |

*Tumour pathology*

The relationship between the grade of malignancy and prognosis in breast carcinoma was demonstrated by Greenough in 1925 and confirmed by Scarff (Patey and Scarff, 1928) and Bloom (1950) and Bloom and Richardson (1957). The effect of host factors has been studied by many workers (McCarty and Mahle, 1921; Black, 1965; Black and Asire, 1969; Black and Speer, 1958, 1960; Black, Kerpe and Speer, 1953; Black, Opler, and Speer, 1954, 1956 and Hamlin, 1968) and in this study the pathology of the tumours was assessed for both these factors using the criteria laid down by Greenough (1925), Patey and Scarff (1928) and Bloom (1950) for the malignancy grading and the method outlined by Hamlin (1968) for the assessment of host factors.

Where slides or blocks were available for study, an assessment of malignancy grading (Bloom, 1950) was made in all cases which had not recieved pre-operative irradiation. A complete assessment of host factors (Hamlin, 1968) could be made only in cases treated by radical mastectomy. For total (simple) mastectomy specimens and partial mastectomy specimens a measure of host defence reaction was attempted by assessing the cellular infiltration in and around the tumour.

Thus the malignancy grading is given as Grade I, II or III in ascending degrees of malignancy. Host defence reaction (H.D.R.) in radical specimens is given as D−, D+ and D++ in ascending degrees of evidence of host reaction; in total (simple) mastectomy specimens and biopsy specimens the measure of host reaction is given as d−, d+ and d++.

*Malignancy grading*

Table XIV gives the malignancy grading correlated with survival in 68 cases treated primarily by radical mastectomy (one intraduct and one colloid being excluded, as this method of grading is inapplicable to these types of breast carcinoma).

TABLE XIV.—*Survival at Follow-up of* 68 *Cases in Malignancy Grades I, II, and III*

| | Malignancy grades | | |
|---|---|---|---|
| No. of cases | I | II | III |
| Total | 5 | 45 | 18 |
| Alive at F.U. at 1 year | 5 | 39 | 16 |
| Alive at F.U. at 2 years | 4 | 36 | 11 |
| Alive at F.U. at 3 years | 4 | 26 | 6 |
| Alive at F.U. at 5 years | 4 | 13 (+6)* | 3 (+2)* |
| Alive at F.U. at 10 years | 2 | 6 (+6)† | 0 (+3)† |

* Cases still alive at follow-up but not followed 5 years.
† Cases still alive at follow-up but not followed 10 years.

The general trend seen here is similar to that found by Bloom (1950) and Bloom and Richardson (1957) and when divided by staging it is clear that the grade of malignancy is closely related to the stage of the disease when first seen, as is the degree of node involvement (Table XV).

TABLE XV.—*Clinical Staging of* 68 *Cases in Malignancy Grades I, II, and III*

| | Stage | | |
|---|---|---|---|
| | Early | Moderate | Late |
| Total No. of cases | 11 | 30 | 27 |
| No. of cases in Grade I | 2 | 2 | 1 |
| No. of cases in Grade II | 6 | 21 | 18 |
| No. of cases in Grade III | 3 | 7 | 8 |

The proportion of cases in Grades II and III is higher in this series of cases under 35 than in Bloom's (1950) series which, of course, includes all ages.

*Host Defence Reaction (H.D.R.) grading*

The correlation between H.D.R. and survival in the patients treated by radical mastectomy is given in the histograms (Fig. 1) and it can be seen that not only do a higher proportion of the cases with a D++ score survive 5 years but the gradient of the fall due to death in the first 3 years is much steeper in

FIG. 1.—Survival of 69 cases treated initially by radical mastectomy subdivided by H.D.R. grading.

those cases with a D— score than those with a D+ and D++ score. These findings agree with those of Hamlin (1968) but again as with the malignancy grading, the proportion of cases in this present series in the poor prognosis D— group, is larger than in Hamlin's (1968) series which included all ages.

When divided by staging of the disease the relationship of H.D.R. to clinical stage is shown (Table XVI).

TABLE XVI.—*Clinical Staging Related to H.D.R.*

| Stage | No. | D— | D+ | D++ |
|---|---|---|---|---|
| Early | 11 | 4 | 3 | 4 |
| Moderate | 30 | 10 | 15 | 5 |
| Late | 28 | 18 | 9 | 1 |
| | 69 | 32 | 27 | 10 |

N.B. Excluding one intraduct in early cases.

*Combined malignancy and H.D.R. gradings*

The 68 radical mastectomy cases in which these gradings are applicable are divided into 5 categories:

| | | |
|---|---|---|
| (1) M++D—: | 28 cases | M++ corresponding to malignancy grades II and III |
| (2) M++D+: | 25 cases | M+ corresponding to malignancy grade I |
| (3) M++D++: | 10 cases | M+ = well differentiated |
| (4) M+D—: | 4 cases | M++ = moderately differentiated and undifferentiated |
| (5) M+D+: | 1 case | |

FIG. 2.—(*a*) Survival of M+ +D— cases treated initially by radical mastectomy. (*b*) Survival of M+ +D+ cases treated initially by radical mastectomy. (*c*) Survival of M+ +D+ + cases treated initially by radical mastectomy.

Survivals for Groups (1), (2) and (3) are shown in three histograms (Fig. 2a, b, and c). Survivals for Groups (4) and (5) are given below:

(4) M+D— : One died within 2 years at 13 months.
One alive at 5 years 4 months.
One alive at 13 years 7 months.
One dead at 11 years 4 months.

(5) M+D+ : One alive 6 years.

Table XVII shows distribution of 68 cases in M and D grades in three clinical stages.

TABLE XVII.—*Clinical Staging Related to M and D Grading*

| Clinical stage | No. | M+ +D— | M+ +D+ | M+ +D+ + | M+D— | M+D+ |
|---|---|---|---|---|---|---|
| Early | 11 | 3 | 2 | 4 | 1 | 1 |
| Intermediate | 30 | 8 | 15 | 5 | 2 | 0 |
| Late | 27 | 17 | 8 | 1 | 1 | 0 |

*Host defence reaction grades of cases treated by mastectomy (not radical)*

Eight cases were treated by total mastectomy as the initial form of treatment (for definition of terms see below). Two cases were intraduct and therefore not given a " d " score. Of the other cases, 3 gave a d— score and none survived 5 years. The other 3 cases gave a d++ score; 1 died at 2 years 5 months, the other 2 were alive at 9 years and 5 years 11 months.

In 13 cases partial mastectomy was performed before radiotherapy. Six cases gave a d— score, all were dead at follow-up, 2 having survived 5 years, dying at 7 years 7 months and 14 years 8 months. Six gave a d+ score; all were dead at follow-up, 2 having survived 5 years 2 months, and 9 years 7 months. One case gave a d++ score and died 7 months after radiotherapy.

*Axillary nodal metastases*

The poorer prognosis which is known to accompany nodal metastases, high grade malignancy and poor host reaction, is again seen in this series. Tables XVIII, XIX and XX give details of survival related to malignancy grading, H.D.R. and nodal metastases.

TABLE XVIII.—*Survival Related to Nodal Metastases in Cases Treated by Radical Mastectomy*

| | No. of cases | No. followed 5 years | No. surviving 5 years | No. followed 10 years | No. surviving 10 years |
|---|---|---|---|---|---|
| Nodes —ve | 16 | 13 | 13 | 6 | 6 |
| Nodes +ve | 54 | 48 | 9 | 44 | 4 |
| Total No. | 70 | 61 | 22 | 50 | 10 |

*Treatment and survival*

Initial treatment varied because many cases were referred by other centres. The cases may however be divided into three categories: radiotherapy, total mastectomy, and radical mastectomy.

*Radiotherapy*

Of the 23 cases in this group, 13 had partial mastectomy before radiotherapy. Partial mastectomy refers to removal of breast lump and surrounding breast tissue. Fifteen cases received surgery after radiotherapy and this was total (3 cases) or radical (11 cases) mastectomy or axillary block dissection (1 case).

Of the 8 cases not receiving subsequent surgery, 2 survived 5 years (both intermediate stage cases). Two of the 3 cases receiving subsequent total mastectomy survived 5 years, one of these dying at 14 years 8 months. Of the 11 cases who had subsequent radical mastectomy, 1 was still alive at 3 years and 2 survived 5 years, 1 of these dying at 10 years 4 months. The one case which had a post radiotherapy axillary dissection died at 7 years and 7 months. Follow-up and survival of radiotherapy cases are depicted in Table XXI. No early stage patient received radiotherapy as the initial treatment.

*Total mastectomy*

Total mastectomy refers to the removal of the breast including its axillary tail, but not the removal of axillary nodes. Of the 8 cases in this group, all

TABLE XIX.—68 *Cases Divided by Malignancy Grading and Nodal Metastases with Follow-up and Survivals at* 1, 2, 3, 5 *and* 10 *Years*

| | | | | No. followed 5 years | | | No. surviving 5 years | | | No. followed 10 years | | | No. surviving 10 years | | |
|---|---|---|---|---|---|---|---|---|---|---|---|---|---|---|---|
| Malig. Grading | Total No. | Nodes − | Nodes + | Total No. | Nodes − | Nodes + | Total No. | Nodes − | Nodes + | Total No. | Nodes − | Nodes + | Total No. | Nodes − | Nodes + |
| | 68 | 14 | 54 | 59 | 11 | 48 | 20 | 11 | 9 | 48 | 4 | 44 | 8 | 4 | 4 |
| I | 5 | 2 | 3 | 5 | 3 | 2 | 4 | 2 | 2 | 2 | 0 | 2 | 2 | 0 | 2 |
| II | 45 | 9 | 36 | 38 | 7 | 31 | 13 | 8 | 5 | 33 | 4 | 29 | 6 | 4 | 2 |
| III | 18 | 3 | 15 | 16 | 1 | 15 | 3 | 1 | 2 | 13 | 0 | 13 | 0 | 0 | 0 |

TABLE XX.—69 *Cases Divided by H.D.R. Grading and Nodal Metastases with Follow-up and Survival at* 1, 2, 3, 5, *and* 10 *Years*

| | | | | No. followed 5 years | | | No. surviving 5 years | | | No. followed 10 years | | | No. surviving 10 years | | |
|---|---|---|---|---|---|---|---|---|---|---|---|---|---|---|---|
| H.D.R. Grading | Total No. | Nodes − | Nodes + | Total No. | Nodes − | Nodes + | Total No. | Nodes − | Nodes + | Total No. | Nodes − | Nodes + | Total No. | Nodes − | Nodes + |
| | 69 | 15 | 54 | 60 | 12 | 48 | 21 | 12 | 9 | 49 | 5 | 44 | 9 | 5 | 4 |
| D− | 32 | 5 | 27 | 28 | 3 | 25 | 7 | 3 | 4 | 26 | 1 | 25 | 4 | 1 | 3 |
| D+ | 27 | 5 | 22 | 24 | 5 | 19 | 9 | 5 | 4 | 19 | 3 | 16 | 4 | 3 | 1 |
| D++ | 10 | 5 | 5 | 8 | 4 | 4 | 5 | 4 | 1 | 4 | 1 | 3 | 1 | 1 | 0 |

TABLE XXI.—*Radiotherapy Cases: Follow-up and Survival Figures at 1, 2, 3, 5, and 10 Years*

| Stage | No. of cases | No. followed 1 yr | No. surviving 1 yr | No. followed 2 yr | No. surviving 2 yr | No. followed 3 yr | No. surviving 3 yr | No. followed 5 yr | No. surviving 5 yr | No. followed 10 yr | No. surviving 10 yr |
|---|---|---|---|---|---|---|---|---|---|---|---|
| Intermediate | 12 | 12 | 11 | 12 | 8 | 12 | 8 | 12 | 6 | 12 | 1 |
| Late | 11 | 11 | 8 | 11 | 4 | 11 | 4 | 10 | 1 | 10 | 1 |
| Total | 23 | 23 | 19 | 23 | 12 | 23 | 12 | 22 | 7 | 22 | 2 |

received post-operative radiotherapy except the 2 intraduct tumours in the early stage, both of which survived 10 years. Two of the 6 cases which had radiotherapy survived 5 years and both of these were in the early stage. Results are shown in Table XXII.

TABLE XXII.—*Total Mastectomy Cases: Follow-up and Survival Figures at 1, 2, 3, 5, and 10 Years*

| Stage | No. of cases | No. followed 1 yr | No. surviving 1 yr | No. followed 2 yr | No. surviving 2 yr | No. followed 3 yr | No. surviving 3 yr | No. followed 5 yr | No. surviving 5 yr | No. followed 10 yr | No. surviving 10 yr |
|---|---|---|---|---|---|---|---|---|---|---|---|
| Early | 5 | 5 | 5 | 5 | 5 | 5 | 4 | 5 | 5 | 3 | 2 |
| Intermediate | 1 | 1 | 1 | 1 | 1 | 1 | 0 | 1 | 0 | 1 | 0 |
| Late | 2 | 2 | 0 | 2 | 0 | 2 | 0 | 2 | 0 | 2 | 0 |
| Total | 8 | 8 | 6 | 8 | 6 | 8 | 4 | 8 | 5 | 6 | 2 |

*Radical mastectomy*

This refers to removal of the breast with pectorales major and minor, and the axillary nodes. Seventy cases fall into this group and their distribution in clinical stages and survival are given in Table XXIII.

TABLE XXIII.—*Radical mastectomy Cases: Follow-up and Survival Figures at 1, 2, 3, 5, and 10 years*

| Stage | No. of cases | No. followed 1 yr | No. surviving 1 yr | No. followed 2 yr | No. surviving 2 yr | No. followed 3 yr | No. surviving 3 yr | No. followed 5 yr | No. surviving 5 yr | No. followed 10 yr | No. surviving 10 yr |
|---|---|---|---|---|---|---|---|---|---|---|---|
| Early | 12 | 12 | 11 | 12 | 11 | 11 | 9 | 9 | 7 | 6 | 4 |
| Intermediate | 30 | 30 | 30 | 30 | 27 | 30 | 20 | 26 | 9 | 21 | 4 |
| Late | 28 | 28 | 21 | 28 | 15 | 28 | 9 | 27 | 6 | 24 | 2 |
| Total | 70 | 70 | 62 | 70 | 53 | 69 | 38 | 62 | 22 | 51 | 10 |

*Early stage*

In the early stage 3 cases had radical mastectomy only (1 an intraduct tumour) and all 3 are alive at 8 years and 15 years (2 cases) later.

Nine cases received radiotherapy which was post-operative in all but 1 case in which there was a delay of 5 months. Three cases were alive at follow-up of less than 5 years; 4 cases, all alive, survived 5 years and 2 of these have survived 10 years.

*Intermediate stage*

Four cases underwent radical mastectomy only. Three of these are alive and have survived 5 years, 2 of which survived 10 years.

Twenty-six cases received radiotherapy which was post-operative in all but 1 case in which there was an interval of 2 years 4 months. Six cases survived 5 years, 1 of which died at 11 years 4 months; the other 5 are alive at periods from 5 years 4 months to 10 years 8 months.

*Late stage*

Two cases underwent radical mastectomy only; both are alive at 7 years 7 months and 10 years 2 months.

Twenty-six cases underwent radical mastectomy and radiotherapy. One case, not followed for 5 years is alive at 3 years 2 months. Four survived 5 years; 1 of these died at 9 years 5 months, and 3 are alive 6 years 1 month, 6 years 5 months and 13 years.

*Treatment related to pathology and survival*

Because pre-operative irradiation alters the histological appearance of both the tumour and the host reaction, cases which received irradiation before mastectomy (partial, total or radical) could not be assessed for malignancy grade or host defence reaction and are therefore excluded from this section. A few cases, *viz.* 3 intraduct carcinomata and 2 " colloid " tumours, *i.e.* types which cannot be given a malignancy grading are also excluded. The 3 intraduct carcinomata were all in the early stage. One of these patients was treated by

TABLE XXV

| Malignancy grade | " d " Grade | HVT+ | HVT− |
|---|---|---|---|
| *Early stage* | | | |
| Grade I —no cases | | | |
| Grade II—no cases | | | |
| Grade III | d− | 1 dead 2 yr 10 mth | — |
| 3 cases | d++ | 1 alive 9 yr | — |
| | | 1 alive 5 yr 11 mth | — |
| *Intermediate stage* | | | |
| Grade I | d+ | 1 dead 9 yr 7 mth | — |
| 1 case | | | |
| Grade II | d− | 1 dead 14 yr 8 mth | — |
| 6 cases | | 1 dead 7 yr 7 mth | |
| | | 1 dead 4 yr 10 mth | |
| | | 1 dead 3 yr 3 mth | |
| | | 1 dead 1 yr 3 mth | |
| | d+ | No cases | — |
| | d++ | 1 dead 2 yr 5 mth | — |
| Grade III | d− | No cases | — |
| 2 cases | d+ | 1 dead 1 yr 3 mth | — |
| | d++ | 1 dead 7 mth | — |
| *Late stage* | | | |
| Grade I No cases | | | |
| Grade II | d− | 1 dead 1 yr 10 mth | |
| 5 cases | | 1 dead 1 yr 4 mth | |
| | d+ | No cases | — |
| | d++ | 3 dead between 3 mth and 4 yr | — |
| Grade III | d− | 1 dead at 7 yr | — |
| 1 case | | | |

TABLE XXIV

| Malignancy grade | D Grade | Nodes+Radiotherapy | Nodes+ No radiotherapy | Nodes— Radiotherapy | Nodes— No radiotherapy |
|---|---|---|---|---|---|
| *Early stage* | | | | | |
| Grade I | D− | 1 dead 13 yr 7 mth | — | — | — |
| 2 cases | D+ | — | — | 1 dead 6 yr | — |
| | D++ | — | — | — | — |
| Grade II | D− | 1 alive 4 yr | — | — | — |
| 6 cases | D+ | 1 dead 9 mth | 1 alive 15 yr 4 mth | — | — |
| | D++ | — | — | 1 alive 3 yr 4 mth | — |
| | | | | 1 alive 10 yr 6 mth | 1 alive 8 yr 2 mth |
| Grade III | D− | 1 dead 3 yr | — | 1 alive 2 yr | — |
| 3 cases | D+ | — | — | — | — |
| | D++ | 1 alive 6 yr | — | — | — |
| *Intermediate stage* | | | | | |
| Grade I | D− | 1 dead 11 yr 4 mth | — | 1 alive 5 yr 4 mth | — |
| 2 cases | D+ | — | — | — | — |
| | D++ | — | — | — | — |
| Grade II | D− | 1 dead 1 yr 5 mth | — | 1 alive 10 yr 8 mth | 1 alive 5 yr |
| 21 cases | | 1 dead 2 yr | | | |
| | | 1 dead 2 yr 5 mth | | | |
| | | 1 dead 2 yr 8 mth | | | |
| | | 1 dead 3 yr | | | |
| | D+ | 1 alive 3 yr 5 mth | — | — | 1 alive 5 yr 10 mth |
| | | 1 alive 3 yr 8 mth | | | |
| | | 1 dead 2 yr 1 mth | | | 1 alive 10 yr 2 mth |
| | | 1 dead 2 yr 7 mth | | | 1 alive 16 yr 5 mth |
| | | 1 dead 4 yr 1 mth | | | |
| | | 1 dead 4 yr 8 mth | | | |
| | | 1 dead 4 yr 10 mth | | | |
| | | 1 dead 4 yr 11 mth | | | |
| | D++ | 1 alive 4 yr 1 mth | 1 dead 3 yr 2 mth | 1 alive 6 yr | — |

| | | | | | |
|---|---|---|---|---|---|
| Grade III. 7 cases | D− | — | — | 1 alive 4 yr 3 mth | — |
| | D+ | 1 alive 7 yr 3 mth<br>1 dead 2 yr 3 mth<br>1 dead 2 yr 5 mth<br>1 dead 4 yr 9 mth | — | — | — |
| | D++ | — | — | — | 1 dead 1 yr 11 mth<br>1 dead 3 yr 3 mth |
| *Late stage* | | | | | |
| Grade I 1 case | D− | 1 dead 13 mth | — | — | — |
| Grade II 18 cases | D− | 1 alive 10 yr 2 mth<br>1 dead 9 yr 5 mth<br>10 dead between 6 mth and 5 yr | — | — | — |
| | D+ | 1 alive 3 yr 2 mth<br>1 alive 6 yr 1 mth<br>1 alive 6 yr 5 mth<br>1 dead 2 yr 1 mth<br>1 dead 2 yr 1 mth<br>1 dead 2 yr 9 mth | — | — | — |
| Grade III 8 cases | D− | 5 dead between 6 mth and 2 yr | — | — | — |
| | D+ | 2 dead under 1 yr | — | — | — |
| | D++ | — | — | — | 1 alive 7 yr 7 mth |

radical mastectomy (alive without signs of recurrence 15 years later) and the other 2 were treated by local mastectomy (both alive with no signs of recurrence at 10 and 16 years). One patient with a colloid tumour (H.D.R. grade D+) had uninvolved nodes and was alive 13 years after radical mastectomy and post-operative radiotherapy. The other patient with a colloid tumour (H.D.R. grade D+) and uninvolved nodes died 5 years 2 months following partial mastectomy and radiotherapy.

Eighty-six cases consisting of 12 partial mastectomy cases, 6 total mastectomy cases and 68 radical mastectomy cases, none of which received pre-operative radiotherapy, are grouped according to malignancy grading (Bloom, 1950) and subdivided according to clinical staging and host defence reaction, H.D.R. (Hamlin, 1968) and treatment, in Tables XXIV and XXV.

### DISCUSSION

McWhirter (1957) states that 10% of breast carcinomata occur under 40 years of age. He calculated that there were approximately 340 new cases of breast carcinoma per million population per year, and assuming that each general practitioner has 2000 patients, and that there are 500 general practitioners per million population, then each general practitioner would see one carcinoma of the breast under 40 years of age every 15 years, approximately twice during his working life. The disease is uncommon under 30 years (de Cholnoky, 1943) and is rare in youth and childhood (Bogen, 1935; McDivitt and Stewart, 1966).

*Age*.—Certain authors (Nathanson and Welch, 1936; Ewing, 1940; Geschickter, 1945; Nohrman, 1949) believe the prognosis to be worse in the under 35 year old group. Certainly the results of this series appear to confirm this. Others (de Cholnoky, 1943; Truscott, 1947; Cade, 1948; MacDonald and Wilcox, 1956; Treves and Holleb, 1958; White, 1960; Watson, 1966) do not think that the prognosis is necessarily worse.

Treves and Holleb (1958) found it impossible to predict results of treatment for any specific age below 35 years on the basis of age alone. Their 5 year clinical cure rates for the age groups, under 26 years, 26 to 30 years, 31 to 34 years were respectively 21%, 43·8% and 40·2%. In this series for similar age groups the 5 year survival figures are 25%, 27% and 41% respectively. It is noteworthy that of 9 cases under 26 years, 2 survived 10 years, both being alive at follow-up. One of these cases had a total mastectomy at the age 24 years for a lump which had been present and increasing in size for 1 year. The lump was an intraduct carcinoma and she was alive 16 years later. The other patient noticed a lump in her breast following trauma when she was 20 years old. Three years later when 3 months pregnant, the lump increased in size and 2 years later when she was 25, a radical mastectomy was carried out. The tumour was of Grade I malignancy, H.D.R. D— and axillary nodes were involved histologically but not detected clinically. Post-operative radiotherapy was given and 5 years later oophorectomy carried out and durabolin given for an axillary recurrence. Eight years later (that is 13 years 7 months after radical mastectomy) there were no signs of recurrence.

*Family history*.—In this series 17 cases gave a family history of breast cancer. In Treves and Holleb's (1958) series about 10% of those under 35 had a family history of breast cancer. Following the pioneer work of Slye (1933) the significance of a family history has been confirmed by many authors (Penrose *et al.*,

1948; Smithers, 1948; Smithers *et al.*, 1952; Treves and Holleb, 1958). " Breast cancer families " have been reported by Wood and Darling (1943), Smithers *et al.* (1952), Oliver (1958) and Stephens *et al.* (1958). Passey (1949) found no evidence of hereditary predisposition to breast cancer whereas Jacobson (1946) found evidence of predisposition to cancer generally but not to breast cancer specifically. On the other hand Penrose *et al.* (1948) and Smithers (1948) found a specific hereditary tendency to breast cancer, but not to cancer generally. Jacobson (1946) observed that breast cancer appeared at an earlier age in patients with a familial predisposition to the disease and Morse (1951) noted that the disease appeared 10 years earlier in daughters whose mothers had the disease. Smithers (1948), though agreeing that a family history is a predisposing factor, found no evidence to suggest that the tumours appear at an earlier age in successive generations.

Three of the cases in this series had been treated by radical at an age at least 20 years younger than when their mothers required similar treatment. In a study of twins one of whom presented with cancer, a greater incidence of cancer has been found in the series of second twins if the twins are monozygotic as opposed to dizygotic (Busk *et al.*, 1948; Smithers *et al.*, 1952). Twins have also been observed to develop breast cancer at similar times, in the same breast and at the identical site in that breast (McFarland and Meade, 1932; Munford and Linder, 1936).

*Marital status.*—Seventy-nine cases (78%) were married and 22 cases (22%) were single, a ratio of approximately 3·6 : 1. In Treves and Holleb's (1958) series, 82% were married and 18% single, a ratio of approximately 4·5 : 1. In the present series 5 and 10 year survivals of the single group were 40% and 22% compared with similar survival times in the married group of 34% and 15% respectively.

Generally the incidence of breast cancer is higher in single than in married women (Smithers *et al.*, 1952; Haagensen, 1956). This is certainly true in the over 40 age-group (Lane-Claypon, 1926; Harnett, 1948; Smithers *et al.*, 1952). Lilienfeld (1956) also noted this increased incidence in single women from 35 to 40 years, but found that under 35 years the rates of single and married women were the same.

*Parity.*—Treves and Holleb (1958) noted that 66% of their cases under 35 years had been pregnant at least once. In this series 57 cases in whom an obstetric history was recorded had been pregnant at least once, although 4 of these did not go to term. The 5 and 10 year survival figures of the nulliparous group were 35% and 20% respectively whilst survival figures for similar periods in the parous group were 31% and 12%. Within the parous group the 5 year survival figures of those who had borne 1, 2, 3 or more children were 27%, 22% and 55% respectively and the 10 year survival figures were 11%, 12% and 14% respectively.

There appears to be less risk of breast cancer developing in women who have borne children (Lane-Claypon, 1926; Bogen, 1935; Clemmesen, 1951; Cappellini *et al.*, 1957; Cutler, 1961). Some figures have shown that the risk decreases with the increasing number of children borne and the incidence in women with 4 or more children is about half that of nulliparous women (Peller, 1940; MacMahon *et al.*, 1968). Recently however, MacMahon (1969) in an international survey has noted that women who have their first child under the age of 20 have only about one-third of the breast cancer risk of those whose first birth is delayed until they are 35 years old, and women whose first birth occurs over the age of 35 have higher breast cancer rates than non-parous women. MacMahon (1969) observed that the protective effect of pregnancy disappears at some point between 25 and 35 years of age and that during pregnancy whilst the output of all oestrogens increases, that of oestriol (non-carcinogenic) increases to a much greater extent than oestrone and oestradiol (carcinogenic fractions). Unfortunately in the present series under study, the ages at which first births occurred were not recorded. Survival rates generally do not differ in nulliparous and parous patients (MacKay and Sellers, 1965; MacMahon *et al.*, 1968) but in this series, the nulliparous women tended to have better 5 and 10 year survival rates. Within the parous group survival does not appear to be related to the number of children born (Peller, 1940; MacMahon *et al.*, 1968). In this series however, 55% of those who had borne 3 or more children survived 5 years.

*Breast feeding.*—Thirty-nine cases (73%) within the parous group had breast fed at least once. Cutler (1961) observed the general opinion that there is an increased incidence of breast cancer in those women who have not borne children or who have not breast fed. The apparent protective effect of parity and breast feeding is noted by Lane-Claypon (1926), Clemmesen (1951), Rennaes and Holan (1953). Lane-Claypon (1926) also suggested that there is an added protection derived from longer duration of breast feeding. The results of an international survey show conclusively that breast feeding is not protective against breast cancer even in areas of the world where prolonged lactation is customary (MacMahon, 1969). In the present series the 5 year survival figure of the group that did not breast feed was 62% and that of the group that did breast feed was 27%.

*Pregnancy and lactation.*—Seventy per cent of breast carcinomas which occur in association with pregnancy are found in women under the age of 30 (Kilgore and Bloodgood, 1929). The incidence of association of breast cancer with pregnancy has been recorded to be between 1% (Robinson, 1965) and 3% (White, 1955).

The obstetrician, however, rarely sees breast cancer complicating pregnancy, the incidence recorded being from 1 in 10,000 pregnancies (Robinson, 1965) to 3 in 10,000 pregnancies (White, 1955) and from 8 in 32,000 deliveries (Nelson, 1956) to 4 in 45,000 deliveries (Power, 1942). Despite this, it is essential that the breasts are examined at regular intervals. Factors which may influence prognosis are:

(1) The breasts are increased in size and a lump is therefore noticed only when large. Fibroadenomata remain easily palpable and mobile but carcinoma, like fibroadenosis, tends to melt into the general breast tissue becoming less significant. Treves and Holleb (1958) found the delay period in the pregnant twice that in the non-pregnant.

(2) The rate of tumour growth may be increased due to high levels of oestrogen (MacMahon, 1969).

(3) Both involvement of regional lymphatic and blood stream dissemination may be increased by the extreme vascularity of the breast in pregnancy.

(4) Tumours occurring during pregnancy are often of high grade malignancy (Bloom, 1955).

Cade (1964) in his assessment, takes into account the effect of the pregnancy on the breast cancer, the effect of the cancer on the pregnancy, the effect of treatment on the cancer and foetus and also the management of the patient with regard to future pregnancies. Cade observed that in the non-pregnant 55% of breast cancers are hormone independent whereas in pregnancy only 10% are hormone independent (Cade, 1964) and provided treatment is adequate, pregnancy has little effect on the course of the lesion. Cade (1964) also noticed regression of breast cancer in cases of spontaneous abortion and he advised termination of pregnancy up to 5 months; if discovered later in pregnancy, he advised Caesarian section or normal delivery. Treves and Holleb (1958) found improved results with termination, as did Cheek (1953) in the first two trimesters. White and White (1956) did not think termination improved results and Adair (1949) advised termination only if axillary nodes were involved.

Recently Peters (1968) outlined her management of breast cancer in pregnancy and lactation:

(1) In the first half of pregnancy the lesion is treated in the conventional manner but no radiotherapy is given and abortion is not advised.

(2) In the latter half of pregnancy, greater caution is advised; if the growth is early or appears less aggressive, the patient is observed and treatment carried out in the early post-partum period and lactation is terminated with oestrogens. With advanced lesions the growth is treated and the pregnancy terminated.

(3) In lactation: this is first suppressed and the breast lesion then treated.

(4) Of subsequent pregnancy, Peters (1968) thinks that the benefits gained outweigh the doubtful benefit of prophylactic castration in the young. Under 35 years she encourages pregnancy after a one year interval following treatment, but over 35 years of age prophylactic castration may be advised when clinically indicated.

Cade (1964) advised against further pregnancies or if this was felt to be too harsh, a delay of at least 5 years before further pregnancy was advised. Harrington (1937) only allowed further pregnancies if the lesion were early and there were no involved nodes but advised a delay of 3 to 5 years. Haagensen and Stout (1943) allowed further pregnancies provided there were no signs of recurrence lest cancer cells may be stimulated by the pregnancy. However, as one million cells can be accommodated in a cubic millimetre, their presence may well be overlooked. White and White (1956) thought that the problem would resolve by natural selection as only those who are fit enough will survive long enough to produce offspring. This again is questionable, as in this series 2 pregnancies were initiated whilst post-radical mastectomy irradiation was being given.

*Delay*.—Sixty per cent of those cases with a delay period of under 1 month had histologically involved axillary nodes compared with 76% of those with a delay period of 3 to 7 months. Five year survival figures were 44% and 26% respectively. Ninety per cent of cases with delay periods of 1 year or more had histologically involved nodes and the 5 year survival figure was 22%.

Nohrman (1949) found that the delay period in younger patients was less than for other ages and 50% of his series of under 35 year old patients presented within 3 months. In the present series, 58% presented within 3 months. With a delay period over 3 months Smithers *et al.* (1952) found a significant drop in survival and concluded that the duration of a tumour is more significant with rapidly growing lesions and that slowly-growing tumours may have a good prognosis even after 1 year. Treves and Holleb (1958) (under 35 year series)

found that cases with a delay period of under 6 months had an axillary node involvement rate of 58·3% with a 42·5% 5 year survival compared with a delay of over 6 months in which 64% had involved axillary nodes and the 5 year survival was 33·8%. Bloom (1965) found that in a series of breast carcinomata from all age groups 64% presented within 6 months and he thought that survival was largely related to the histological type of growth. Eberbach (1949) suggested that the rate of growth of the tumour is the deciding factor, and as Kraus (1953) also observed, presentation is earlier with the more malignant tumours than with the less malignant ones which grow more slowly, have a longer delay period, and a better prognosis. White (1960) found that with a delay over 6 months, there was a greater incidence of involved nodes although many cases did well; presumably in these cases relatively slow growth was accompanied by good host reaction.

Strax *et al.* (1969) have shown that with earlier diagnosis, more patients will be treated in the early stage, and there should therefore be improved results. They found that repeated screening by both clinical examination and mammography resulted in a greater number of carcinomata being detected than by either method alone. In the control group (*i.e.* those not subjected to screening) 56% of patients with breast carcinoma had evidence of axillary node involvement compared with 35% of those patients found to have carcinoma on initial screening and 19% of those diagnosed on subsequent screenings.

McSwain and Coles (1947) and Berkson *et al.* (1957) found that over the years there had been an increase in the number of patients with uninvolved nodes with a corresponding improvement in survival.

Park and Lees (1951) and McKinnon (1954) suggested that earlier diagnosis does not improve the results of surgical treatment and imply that no treatment of the disease by any method prolongs the patient's life.

*Clinical presentation.*—Treves and Holleb (1958) observed that only 1% of their cases was discovered in a routine medical examination. In this series no case was so discovered.

It has been suggested that when a breast cancer becomes clinically apparent it is in the final quarter of its life (Collins *et al.*, 1956). Strax *et al.* (1969) however, have shown that less malignant and less extensive lesions with more favourable prognoses have been detected by screening methods with consequent reduction of the delay period.

Regular self-examination or attendance at a special breast clinic may help towards early diagnosis.

*Clinical stage.*—The clinical stage is an assessment of the extent of the disease. There have been many methods of staging and at present the T.N.M. system is internationally recognized. Difficulties and inaccuracies arise from varying methods of tumour measurement (see below) and assessment of regional lymph nodes and detection of metastases. In this series cases were classified on a T.N.M. basis. Although the staging was approximate, the prognosis, as in all series, is closely related to the clinical stage with a relatively high 3 and 5 year survival of cases in the early stage (81% and 78% respectively) and a poorer survival in the intermediate and late stages (intermediate 65% and 38%, late 32% and 18% respectively). Similarly within each stage the proportion of cases surviving 10 years is much greater in the early stage (67%) than the proportion of cases surviving 10 years in the intermediate (14%) and late stage (8%).

*Size.*—Approximately half the tumours were 2–5 cm. in their greatest diameter. A correlation between tumour size and prognosis is found in this series; the smaller the tumour, the better the prognosis. This confirms results of Harrington (1946), Rennaes (1960), and Robbins (1962). Treves and Holleb (1958) thought that size alone was of prognostic significance regardless of axillary node involvement and that the 5 year clinical cure rate diminished as tumour size increased. White (1960) noted that with tumours less than 5 cm., the smaller the size the better the prognosis. Further analysis of the cases studied by Hamlin (1968) has shown a correlation between size and prognosis (as yet unpublished). However as Cutler (1961) observed, some small primary tumours produce massive secondaries and size alone is not always an index of curability.

*Site.*—Distribution of cases in the various sites in this series is similar to that seen in all ages but survival appears approximately equal for all sites. Treves and Holleb (1958) found the tumour site to have little or no influence on the clinical cure rate.

Most authors, however, working with cases of all ages, found a worse prognosis with inner quadrant tumours especially in early stage growths (Haagensen and Stout, 1943; Truscott, 1947; Nohrman, 1949; Handley, 1951; Urban, 1959). Conversely in 1951 Haagensen and Stout published a series in which patients with tumours in the inner quadrants had the better prognosis.

*Pathology.*—In the series of 273 cases from all age groups published by Hamlin in 1968, several points relating to the pathology of the tumours were noted:

(1) Well differentiated carcinomas of Grade I malignancy had a good prognosis with a 50% 15 year survival both in the presence and absence of host reaction.

(2) The prognosis of cases with tumours of malignancy Grades II and III was closely related to the presence or absence of nodal metastases and to the host defence reaction. It was also noted that these two factors were related to each other.

(3) There was a higher incidence of the histological types of tumours which have a relatively poor prognosis in patients who developed breast carcinoma during the reproductive years of life, *i.e.* before the age of 45, and the prognosis associated with any one histological group is less good in this age group than in the age group in the premenopausal years, *i.e.* 45 to 55 years.

The analyses in this series show the same general trend. Of the group of cases with malignancy Grade I tumours, only one case which was in the late clinical stage, died under 5 years. The long survival of the other cases (all still alive) is unrelated to the H.D.R.

In only 16 (23%) of the 70 patients treated by radical mastectomy were the axillary nodes found histologically free of metastases. All these cases are alive at follow-up at from 2 years to 16 years after radical mastectomy.

Of 63 cases with malignancy Grades II and III tumours, 12 had uninvolved lymph nodes; in all but 4 cases the absence of axillary metastases was accompanied by some evidence of host reaction and all but 2 cases are alive at follow-up. Long-term survival of cases with these more malignant tumours, with or without invaded lymph nodes, was certainly more frequent among those cases in which a host reaction was present (Fig. 2).

Long-term survival in the Hamlin (1968) series was found to be associated with tumours of Grade I malignancy, and with tumours of Grades II and III malignancy, which were accompanied by a measurable degree of host reaction.

The proportion of cases in the present series with Grade I malignancy tumours, 5 out of 101, is much less than in the Bloom and Richardson series published in 1957 (362 out of 1,409) and in Hamlin's series (1968) (61 out of 273). Both of these earlier series were drawn from cases of all age groups.

The proportion of cases with tumours of malignancy Grades II and III in the three D grades in the Hamlin series was D—, 32·2%; D+, 41·6%; and D++, 25·5%. In the present series, the proportion of cases in the poorer prognosis group giving a D— score is much greater, *viz.* D— 41%, D+ 37% and D++ 15%.

Because a high proportion of the cases in the breast series have not yet been followed up for 10 years, it is not possible to compare the prognosis in each of the separate M and D groups with those in the larger series analysed by this method (Hamlin, 1968), but it is clear that a higher proportion of cases in this young age group series have tumours with histological appearances that have been found to be associated with a poor prognosis (Hamlin, 1968).

*Treatment and survival.*—Probably no subject arouses as much controversy as the treatment of carcinoma of the breast. A recent survey of the Fellows of the Association of Surgeons of Great Britain and Ireland revealed the diversity of opinions on methods of treatment for a particular stage of the disease (*British Journal of Surgery*, 1969). In this country, controlled clinical trials are at present being conducted to assess the value of various treatments. Despite advances, the death rate over the last 30 years has remained static (Adams and Spicer, 1965; Cutler, 1966). In most series for all ages the 5 year survival figure is 50% and the 10 year 30% (Forrest, 1969). In this series of under 35 year old patients, 5 and 10 year survival figures are 36% and 18% respectively.

Most would agree that total or radical mastectomy with or without radiotherapy are the main forms of treatment for cases in Stages I and II (corresponding with early and intermediate stages in this series), but that for cases in Stages III and IV radiotherapy is to be preferred. In this series radiotherapy as the primary treatment was given only to intermediate (12) and late (11) stage cases. Understandably the 5 year survival figure of this group (32%) was less than that of the 8 cases treated primarily by total mastectomy (50%) for in this latter group 5 cases were in the early stage, 2 being intraduct tumour cases. A very large proportion of the 70 cases for whom radical mastectomy was the primary treatment received subsequent radiotherapy. Of the 61 cases (87%) which did receive subsequent radiotherapy, 14 cases survived 5 years, these being alive at follow-up from 5 years 4 months to 13 years 7 months except for 2 cases that died at 9 years 5 months and 11 years 4 months. Thirteen per cent of the cases treated primarily by radical mastectomy did not receive subsequent radiotherapy; of these only 1 died under 5 years, the other 8 are all alive at follow-up from 5–16 years after radical mastectomy. All but 1 of these cases had negative nodes and all but 1 other case showed an H.D.R. of D+ or D++. A number of authorities have questioned the value of post-operative radiotherapy (Hall and Bagby, 1938; de Cholnoky, 1943; Treves and Holleb, 1958). Easson (1968) reported identical survival of Stages I and II radical mastectomy cases with and without post-operative irradiation. Bond (1967) thinks irradiation is contra-indicated in those cases in which the nodes are negative. A number of treatment trials for breast carcinoma are now in progress and when these are complete it may be possible to assess the value of post-operative irradiation in the treatment of breast carcinoma. It is important, however, in such trials to have detailed assessment of all factors which have been shown to have some influence on prognosis so that a clear picture may be gained of the effect which different forms of treatment are having.

Three points with regard to clinical evaluation require elaboration:

(1) The clinical assessment of node involvement is notoriously inaccurate.

(2) The measurement of tumour size by callipers may be misleading. A "scirrhous" tumour surrounded by adenosis may measure 6 cm. T3 in clinical examination, but when excised is found to contain a 2 cm. tumour T1.

Thus on clinical grounds this would be assigned to an advanced stage (III) and treated by radiotherapy rather than surgery.

(3) The T.N.M. staging sizes especially T1 $<$ 2 cm. to T2 5 cm. represents too great a difference because the prognosis for a 2·5 cm. T2 is quite different from a 5 cm. tumour T2.

In all but the most advanced cases when a drill or a needle biopsy only is necessary to confirm the clinical diagnosis, the lump must be removed for frozen section examination.

Under anaesthesia a more accurate evaluation of tumour size and node involvement may be made and the breast lump excised with an adequate margin of normal tissue measured and submitted to frozen section (for immediate diagnosis) and paraffin section (for tumour characterization). It is suggested that the treatment plan should at this stage be based on the size of the tumour, the malignancy grading and the presence and degree of host reaction.

### CONCLUSIONS

In this series prognosis appears to be related to the delay period, clinical stage, size of tumour, the histological appearances of the tumour, and presence or absence of axillary metastases.

In the histological appearances of the tumour, both the grade of malignancy of the tumour and the host reaction to it appear to be related to prognosis.

In comparison with groups of patients drawn from all age groups, a smaller proportion of patients in this age group have tumours of malignancy Grade I and of those with tumours of malignancy Grades II and III a smaller proportion exhibit a good host reaction.

We are grateful to the many pathologists of referring hospitals for their co-operation in supplying histological material, and to the surgeons and radiotherapists of the Royal Marsden Hospital and referring hospitals for allowing us to study cases admitted under their care.

We wish to thank Mr. C. H. Chadwin and Miss D. Bower for technical assistance, Miss L. Pegus for the illustrations and Miss M. Craddock for secretarial assistance.

### REFERENCES

ADAIR, F. E.—(1949) *Ann. R. Coll. Surg.*, **4**, 360.

ADAMS, M. J. T. AND SPICER, C. C.—(1965) *Lancet*, ii, 732.

BERKSON, J., HARRINGTON, S. W., CLAGETT, O. T., KIRKLIN, J. W., DOCKERTY, M. B. AND MCDONALD, J. R.—(1957) *Staff Meet. Mayo Clin.*, **32**, 645.

BLACK, M. M.—(1965) *Prog. clin. Cancer*, **1**, 26.

BLACK, M. M. AND ASIRE, A.—(1969) *Cancer, N.Y.*, **23**, 251.
BLACK, M. M., KERPE, S. AND SPEER, F. D.—(1953) *Am. J. Path.* **29**, 505.
BLACK, M. M., OPLER, S. R. AND SPEER, F. D.—(1954) *Surgery, Gynec. Obstet.*, **98**, 725.—(1956) *Surgery, Gynec. Obstet.*, **102**, 599.
BLACK, M. M. AND SPEER, F. D.—(1958) *Surgery Gynec. Obstet.*, **106**, 163.—(1960) *Surgery Gynec. Obstet.*, **110**, 477.
BLOOM, H. J. G.—(1950) *Br. J. Cancer*, **4**, 259, 347.—(1955) *Rep. Br. Emp. Cancer Campn*, **33**, 30.—(1965) *Br. J. Cancer*, **19**, 228.
BLOOM, H. J. G. AND RICHARDSON, W. W.—(1957) *Br. J. Cancer*, **11**, 359.
BOGEN, R.—(1935) *Am. J. publ. Hlth*, **25**, 245.
BOND, W. H.—(1967) reported in *World Medicine*, October 17, p. 68.
BRITISH JOURNAL OF SURGERY—(1969) Breast Cancer Symposium, **56**, 782.
BUSK, T., CLEMMESEN, J. AND NIELSEN, A.—(1948) *Br. J. Cancer*, **2**, 156.
CADE, S.—(1948) *J. Am. med. Ass.*, **138**, 1083.—(1964) *J. Obstet. Gynaec. Br. Commonw.*, **71**, 341.
CAPELLINI, E., GUFFANTI, A. AND MONTINI, T.—(1957) *Rass. ital. Chir. Med.*, **6**, 1.
CHEEK, J. H.—(1953) *Archs Surg.*, **66**, 664.
CLEMMESEN, J.—(1951) Report on Oxford Symposium 1950. *Acta Un. int. Cancr.*, **7**, 24.
COLLINS, V. P., LOEFFLER, R. K. AND TIVEY, H.—(1956) *Am. J. Roentg.*, **76**, 988.
CUTLER, M.—(1961) 'Tumours of the Breast'. London (Pitman Medical Publishing Co. Ltd.), pp. 152, 246.
CUTLER, S. J.—(1966) in 'Clinical Evaluation in Breast Cancer', edited by J. L. Hayward and R. D. Bulbrook. New York (Academic Press), p. 215.
DARGENT, M. AND MAYER, M.—(1948) *Presse méd.*, **56**, 561.
DE CHOLNOKY, T.—(1943) *Surgery, Gynec. Obstet.*, **77**, 55.
EASSON, E. C.—(1968) in 'Factors Influencing Prognosis of Breast Cancer', edited by A. P. M. Forrest and P. B. Kunkler. Edinburgh (Livingstone), p. 118.
EBERBACH, C. W.—(1949) *Wis. med. J.*, **48**, 132.
EWING, J.—(1940) 'Neoplastic Diseases; a Treatise on Tumours', 4th edition. Philadelphia (W. B. Saunders Co.).
FORREST, A. P. M.—(1969) in 'Recent Advances in Surgery', edited by Selwyn Taylor. London (Churchill), p. 84.
GESCHICKTER, C. F.—(1945) 'Diseases of the Breast', 2nd edition. Philadelphia (Lippincott), p. 404.
GREENOUGH, R. B.—(1925) *J. Cancer Res.*, **9**, 453.
GROSS, S. W. A.—(1880) 'A Practical Treatise on Tumours of the Mammary Gland; Embracing their Histology, Pathology, Diagnosis, and Treatment'. New York (D. Appleton & Co.), p. 146.
HAAGENSEN, C. D.—(1956) 'Diseases of the Breast'. Philadelphia (Saunders).
HAAGENSEN, C. D. AND STOUT, A. P.—(1943) *Ann. Surg.*, **118**, 859.—(1951) *Ann. Surg.*, **134**, 151.
HALL, N. AND BAGBY, J. W.—(1938) *J. Am. med. Ass.*, **110**, 703.
HAMLIN, I.—(1968) *Br. J. Cancer*, **22**, 383.
HANDLEY, R. S.—(1951) *Proc. R. Soc. Med.*, **45**, 565.
HARNETT, W. L.—(1948) *Br. J. Cancer*, **2**, 212.
HARRINGTON, S. W.—(1937) *Trans. Am. surg. Ass.*, **55**, 209.—(1946) *Surgery, St. Louis*, **19**, 154.
JACOBSON, O.—(1946) 'Heredity in Breast Cancer. A Genetic and Clinical Study of 200 Probands'. London (H. K. Lewis & Co., Ltd.).
KILGORE, A. R. AND BLOODGOOD, J. C.—(1929) *Archs Surg.*, **18**, 2079.
KRAUS, A. S.—(1953) *Surgery, Gynec. Obstet.*, **96**, 545.
LANE-CLAYPON, J. E.—(1926) Ministry of Health Reports on Public Health and Medical Subjects, No. 32. London (H.M. Stationery Office).
LILIENFIELD, A. M.—(1956) *Cancer, N.Y.*, **9**, 927.

McCarty, W. C. and Mahle, A. E.—(1921) *J. Lab. clin. Med.*, **6**, 473.
McDivitt, R. W. and Stewart, F. W.—(1966) *J. Am. med. Ass.*, **195**, 388.
MacDonald, I. and Wilcox, N. E.—(1956) *Cancer, N.Y.*, **9**, 281.
McFarland, J. and Meade, T. S.—(1932) *Am. J. med. Sci.*, **184**, 66.
MacKay, E. N. and Sellers, A. H.—(1965) *Can. med. Ass. J.*, **92**, 647.
McKinnon, N. E.—(1954) *Lancet*, i, 251.
MacMahon, B., List, N. D. and Eisenberg, H.—(1968) in ' Prognostic Factors in Breast Cancer ', edited by A. P. M. Forrest and P. B. Kunkler. Edinburgh (Livingstone), p. 56.—(1969) Progress Report—International Collaborative Study of Breast Cancer, p. 12.
McSwain, B. and Coles, J. H.—(1947) *Cancer, N.Y.*, **10**, 469.
McWhirter, R.—(1957) *J. Fac. Radiol.*, **8**, 220.
Morse, D. P.—(1951) *Cancer, N.Y.*, **4**, 745.
Munford, S. E. and Linder, H.—(1936) *Am. J. Cancer*, **28**, 393.
Nathanson, I. T. and Welch, C. E.—(1936) *Am. J. Cancer*, **28**, 40.
Nelson, H. M.—(1956) *Rocky Mount. med. J.*, **53**, 287.
Nohrman, B. A.—(1949) *Acta radiol.*, Suppl. 77, p. 1.
Oliver, C. P.—(1958) *Ann. N.Y. Acad. Sci.*, **71**, 1198.
Park, W. N. and Lees, J. C.—(1951) *Surgery Gynec. Obstet.*, **93**, 129.
Passey, R. C.—(1949) *Rep. Br. Emp. Cancer Campn*, **27**, 168.
Patey, D. H. and Scarff, R. W.—(1928) *Lancet*, i, 801.
Peller, S.—(1940) *Surgery Gynec. Obstet.*, **71**, 181.
Penrose, L. S., Mackenzie, H. J. and Karn, M. N.—(1948) *Br. J. Cancer*, **2**, 168.
Peters, V.—(1968) in ' Prognostic Factors in Breast Cancer ', edited by A. P. M. Forrest and P. B. Kunkler. Edinburgh (Livingstone).
Power, H. A.—(1942) *Penn. med. J.*, **45**, 1049.
Rennaes, S. and Holan, L.—(1953) *Nord. Med.*, **50**, 967.
Rennaes, S.—(1960) *Acta chir. scand.*, Suppl. 166.
Robbins, G. F.—(1962) *Acta Un. int. Cancr.*, **18**, 864.
Robinson, D. W.—(1965) *Am. J. Obstet. Gynec.*, **92**, 658.
Slye, M.—(1933) *Am. J. Cancer*, **18**, 535.
Smithers, D. W.—(1948) *Br. J. Cancer*, **2**, 163.
Smithers, D. W., Rigby-Jones, P., Galton, D. A. G. and Payne, P. M.—(1952) *Br. J. Radiol.*, Suppl. 4, p. 3.
Stephens, F. E., Gardner, E. J. and Woolf, C. M.—(1958) *Cancer, N.Y.*, **11**, 967.
Strax, P., Venet, L. and Shapiro, S.—(1969) *Cancer, N.Y.*, **23**, 875.
Taylor, G. W. and Wallace, W. R. H.—(1947) *Surg. Clins N. Am.*, **27**, 1151.
T.N.M. Classification of Malignant Tumours—(1958) Union Internationale contre le Cancer, Geneva, p. 39.
Treves, N. and Holleb, A. I.—(1958) *Surgery Gynec. Obstet.*, **107**, 271.
Truscott, B. M.—(1947) *Br. J. Cancer*, **1**, 129.
Urban, J. A.—(1959) *Cancer, N.Y.*, **12**, 14.
Watson, T. A.—(1966) *Am. J. Roentg.*, **96**, 547.
White, T. T.—(1955) *Am. J. Obstet. Gynec.*, **69**, 1277.
White, T. T.—(1955) *Surgery Gynec. Obstet.*, **100**, 661.
White, T. T.—(1960) *NW. Med., Seattle*, **59**, 218.
White, T. T. and White, W. C.—(1956) *Ann. Surg.*, **144**, 384.
Wood, D. A. and Darling, H. H.—(1943) *Cancer Res.*, **3**, 509.